# Supplementary figures and images for: C. elegans toxicant responses vary among genetically diverse individuals
Source: Toxicology. Author manuscript; Available in PMC 2022 Oct 16. (PMC9573778; doi:10.1016/j.tox.2022.153292)

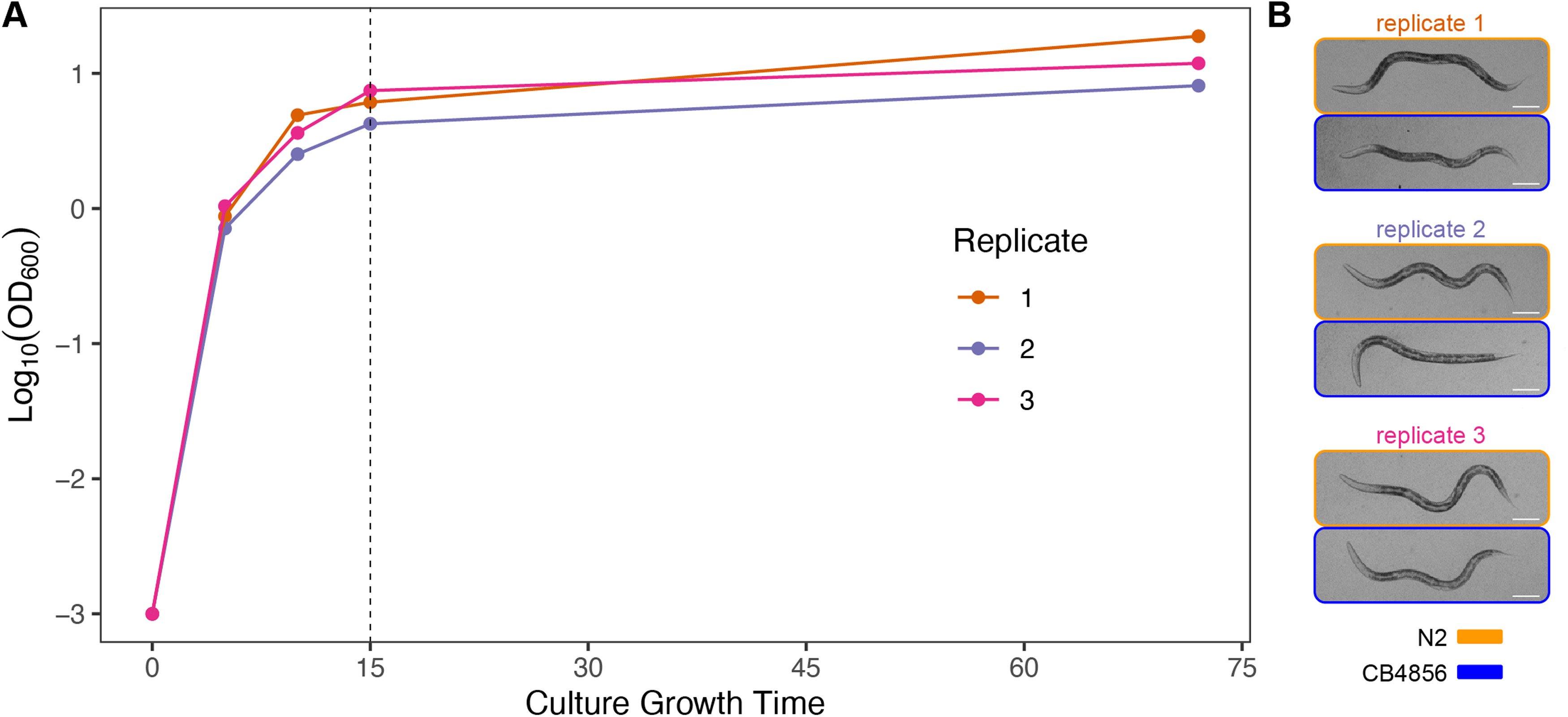

Supplement: SupFig1 [file NIHMS1838727-supplement-SupFig1.jpg]

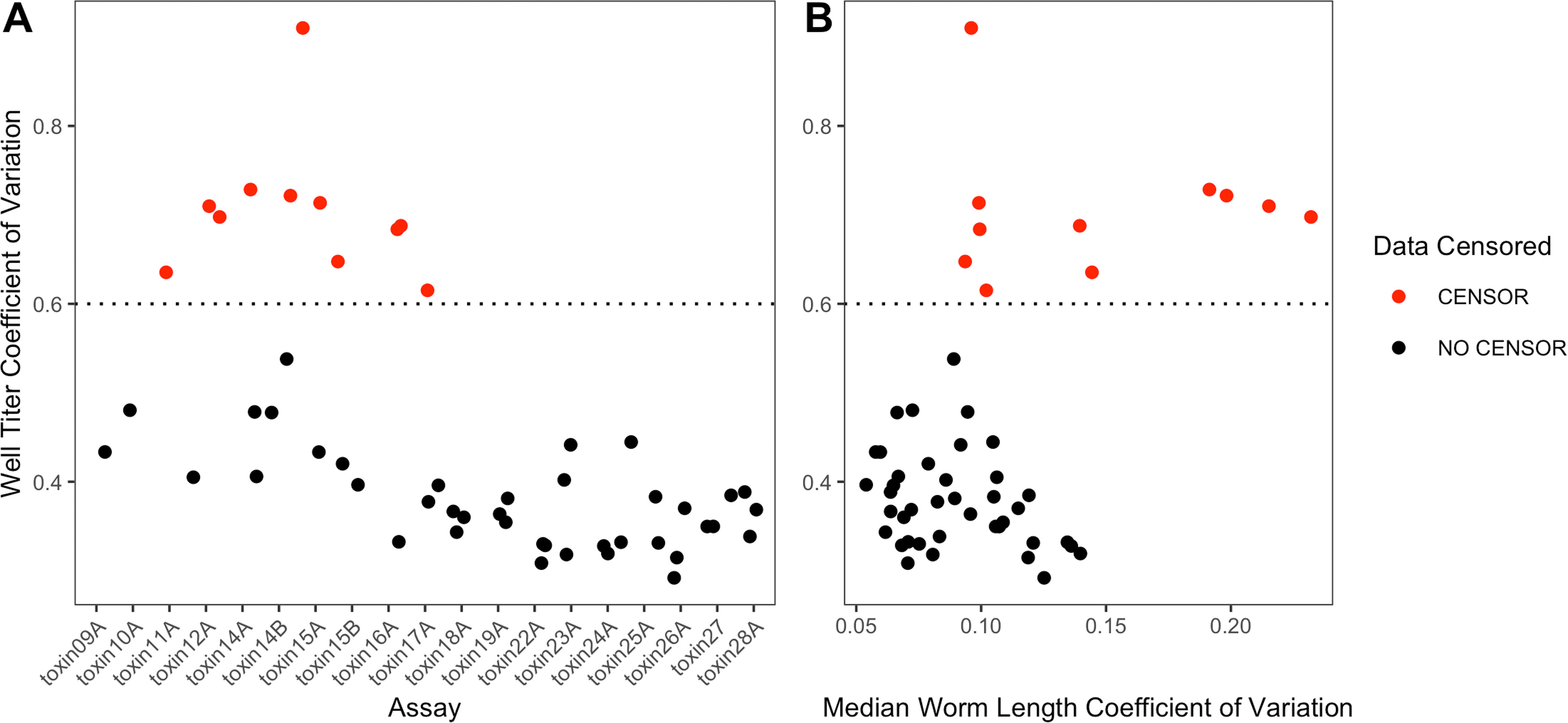

Supplement: SupFig2 [file NIHMS1838727-supplement-SupFig2.jpg]

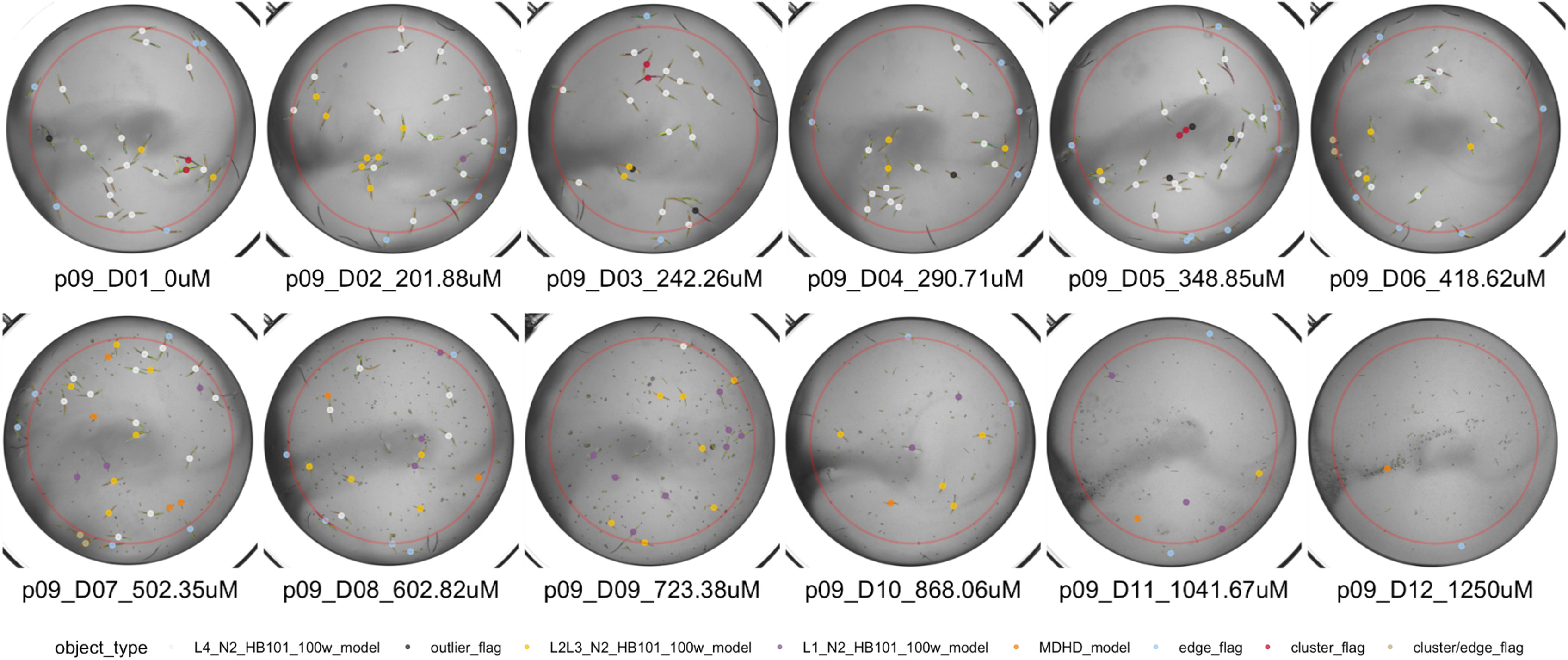

Supplement: SupFig3 [file NIHMS1838727-supplement-SupFig3.jpg]

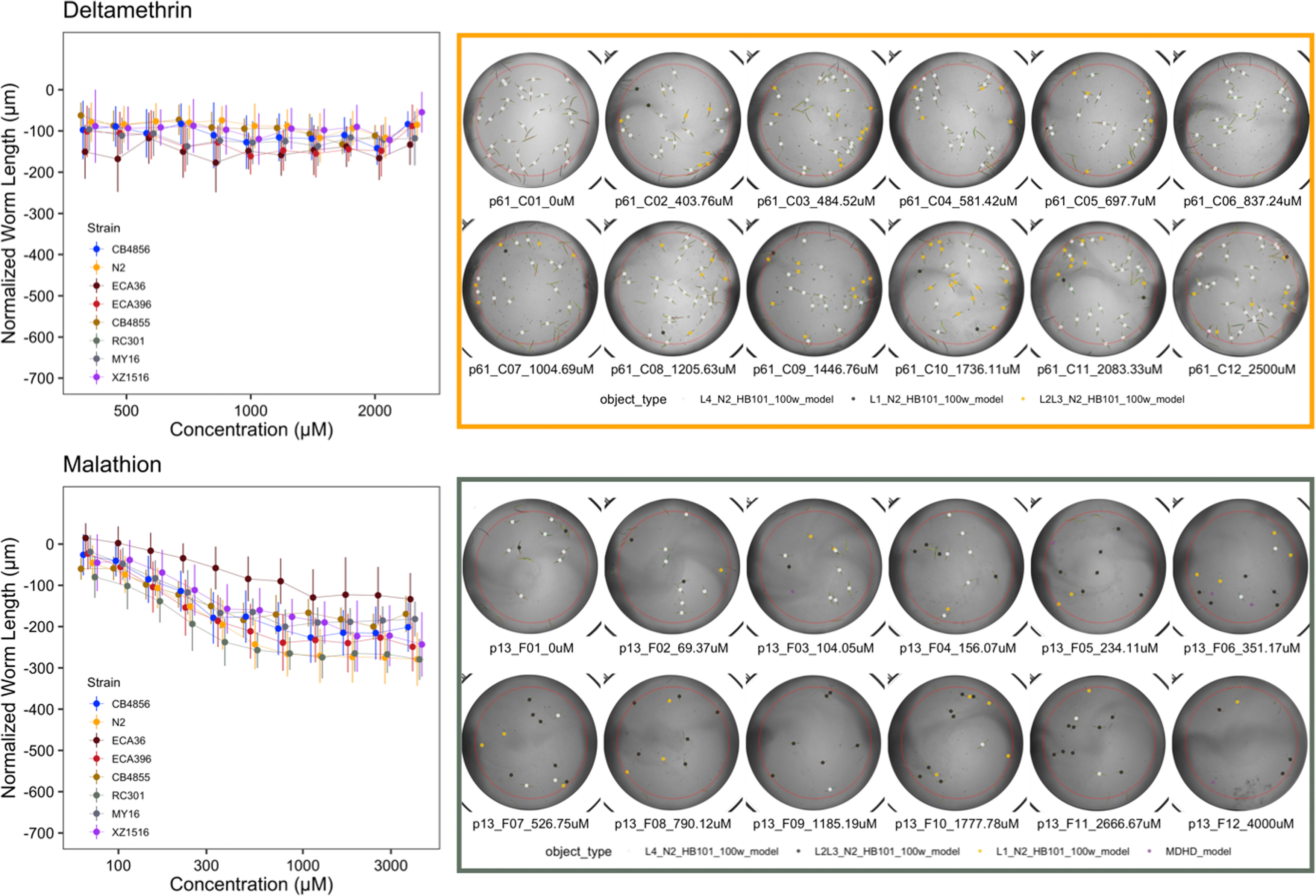

Supplement: SupFig4 [file NIHMS1838727-supplement-SupFig4.jpg]

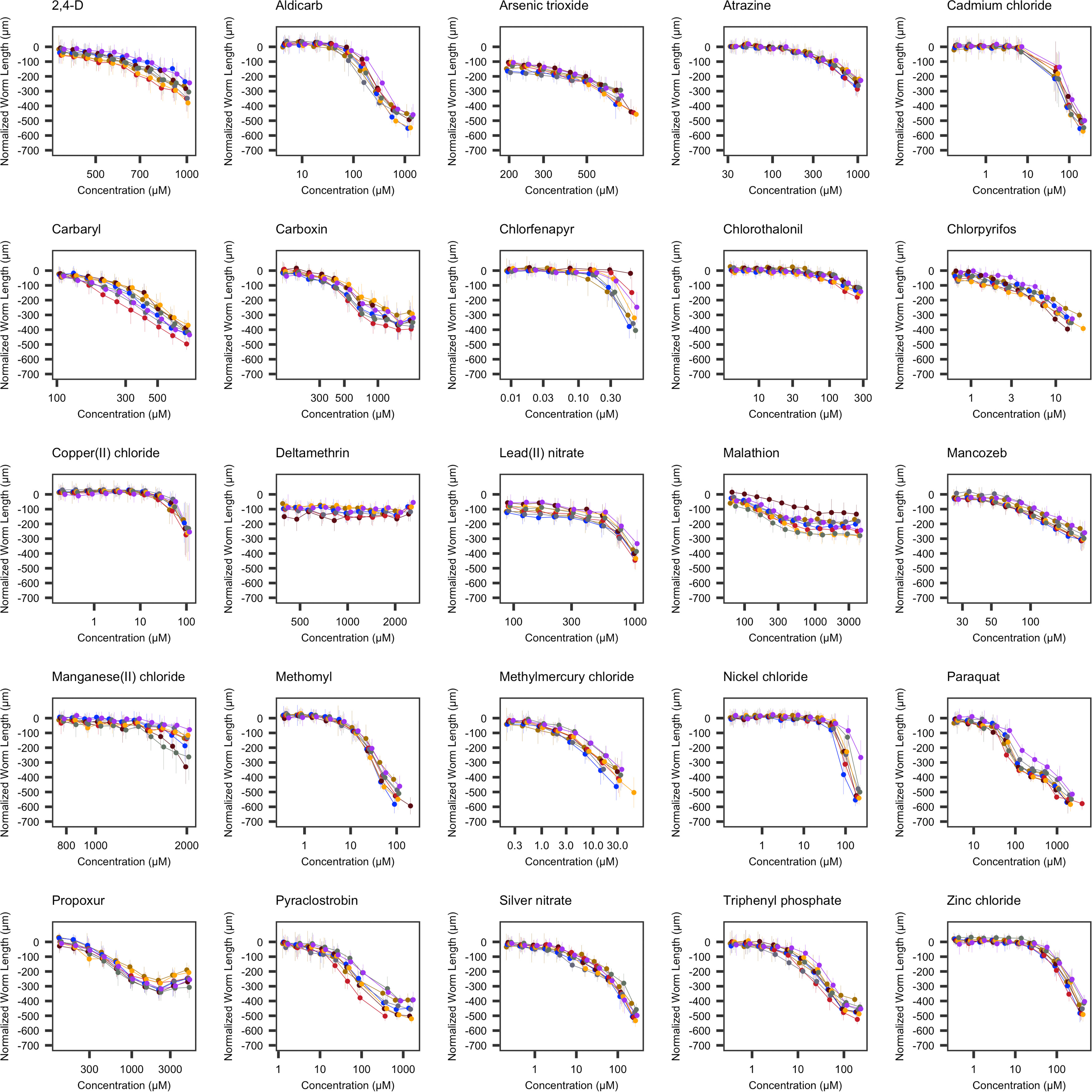

Supplement: SupFig5 [file NIHMS1838727-supplement-SupFig5.jpg]

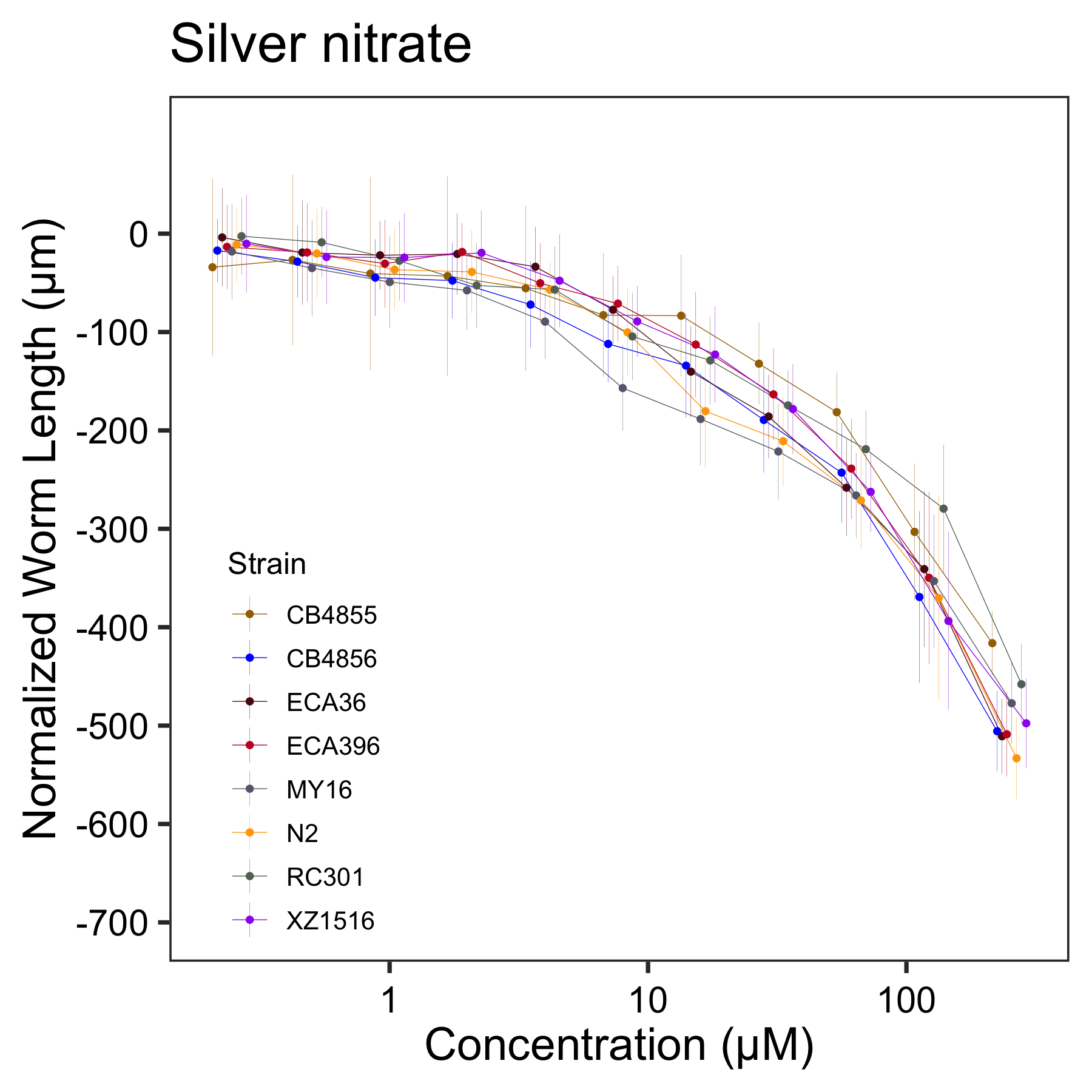

Supplement: SupFigs6-30 [file NIHMS1838727-supplement-SupFigs6-30.zip › mmc7/supp.fig.10.png]

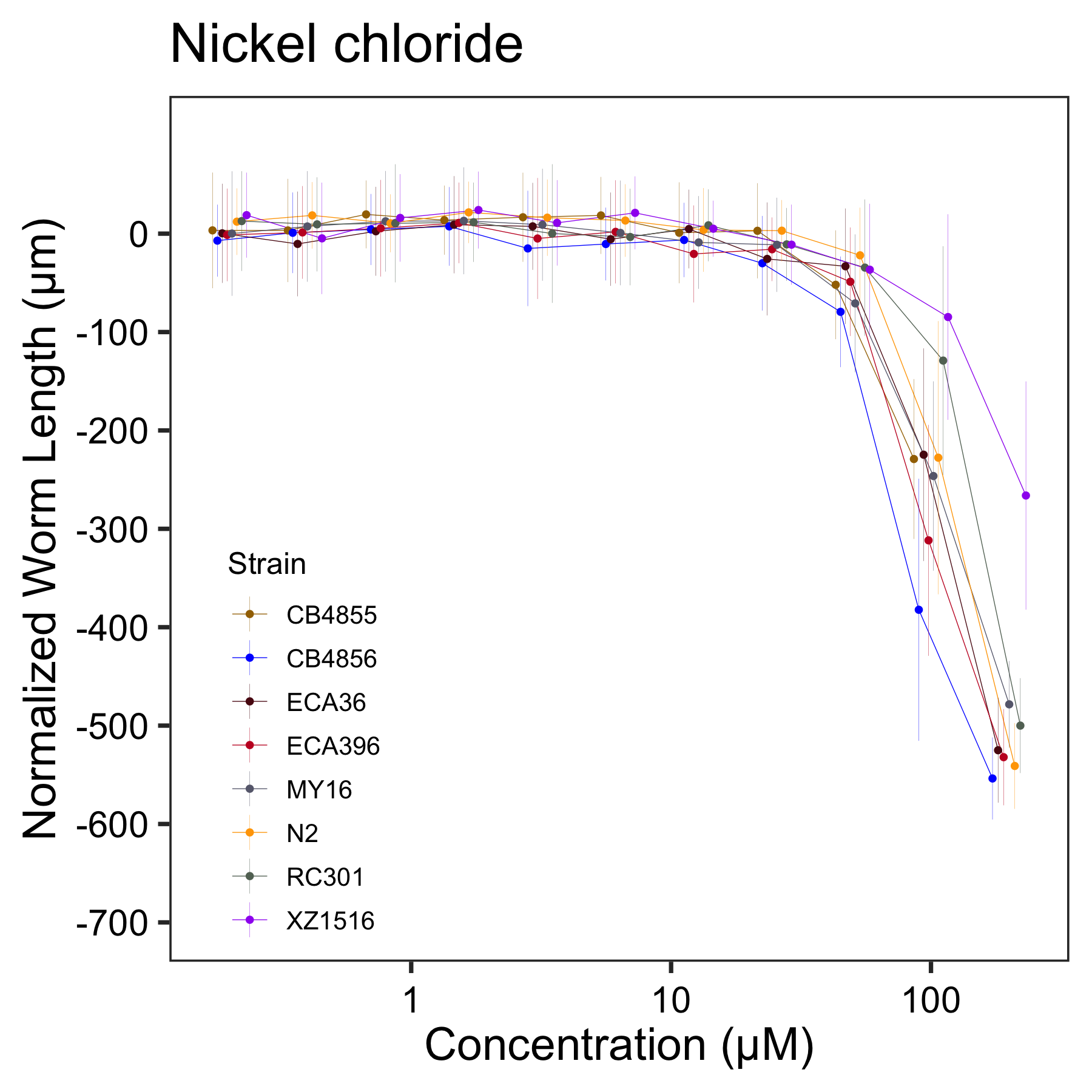

Supplement: SupFigs6-30 [file NIHMS1838727-supplement-SupFigs6-30.zip › mmc7/supp.fig.11.png]

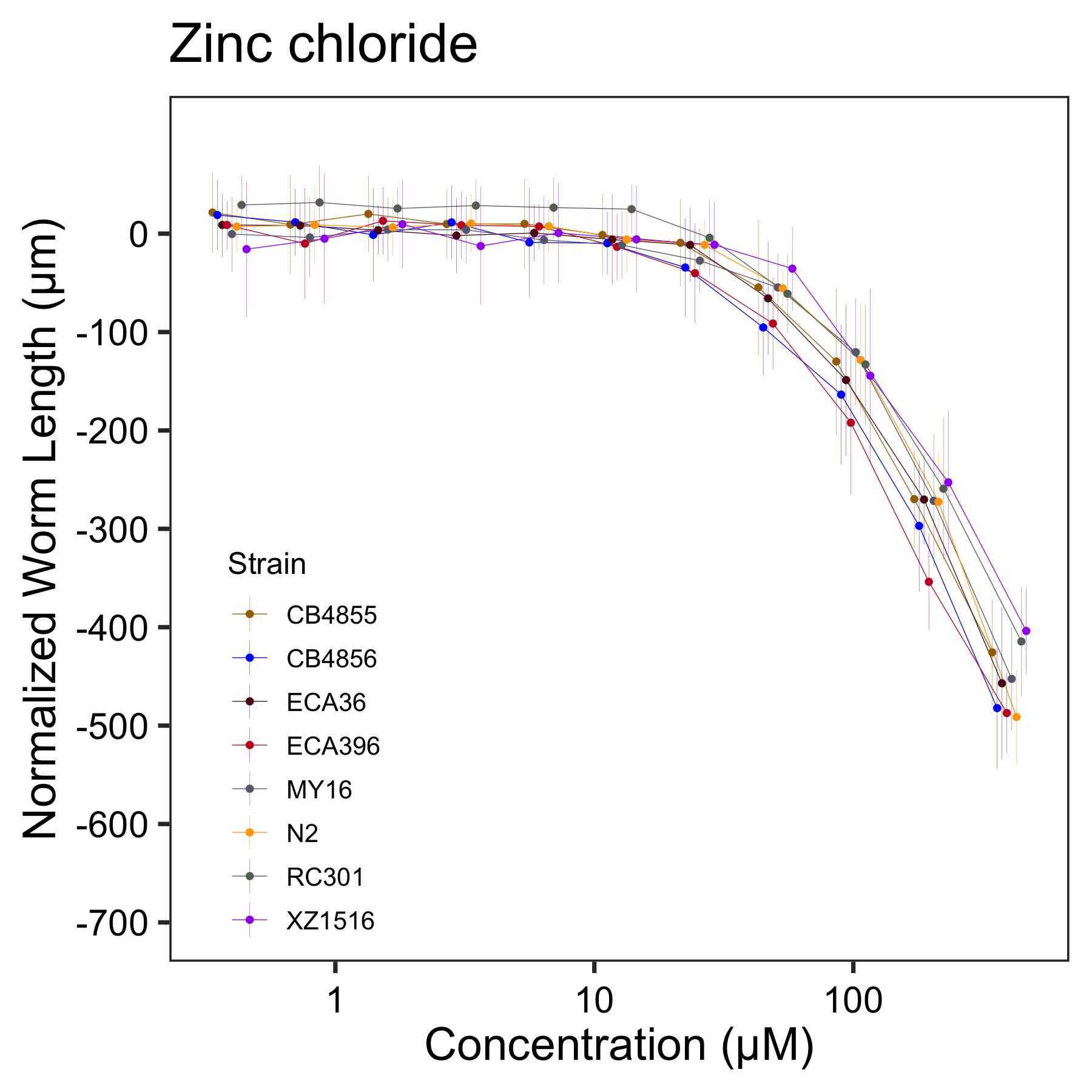

Supplement: SupFigs6-30 [file NIHMS1838727-supplement-SupFigs6-30.zip › mmc7/supp.fig.12.png]

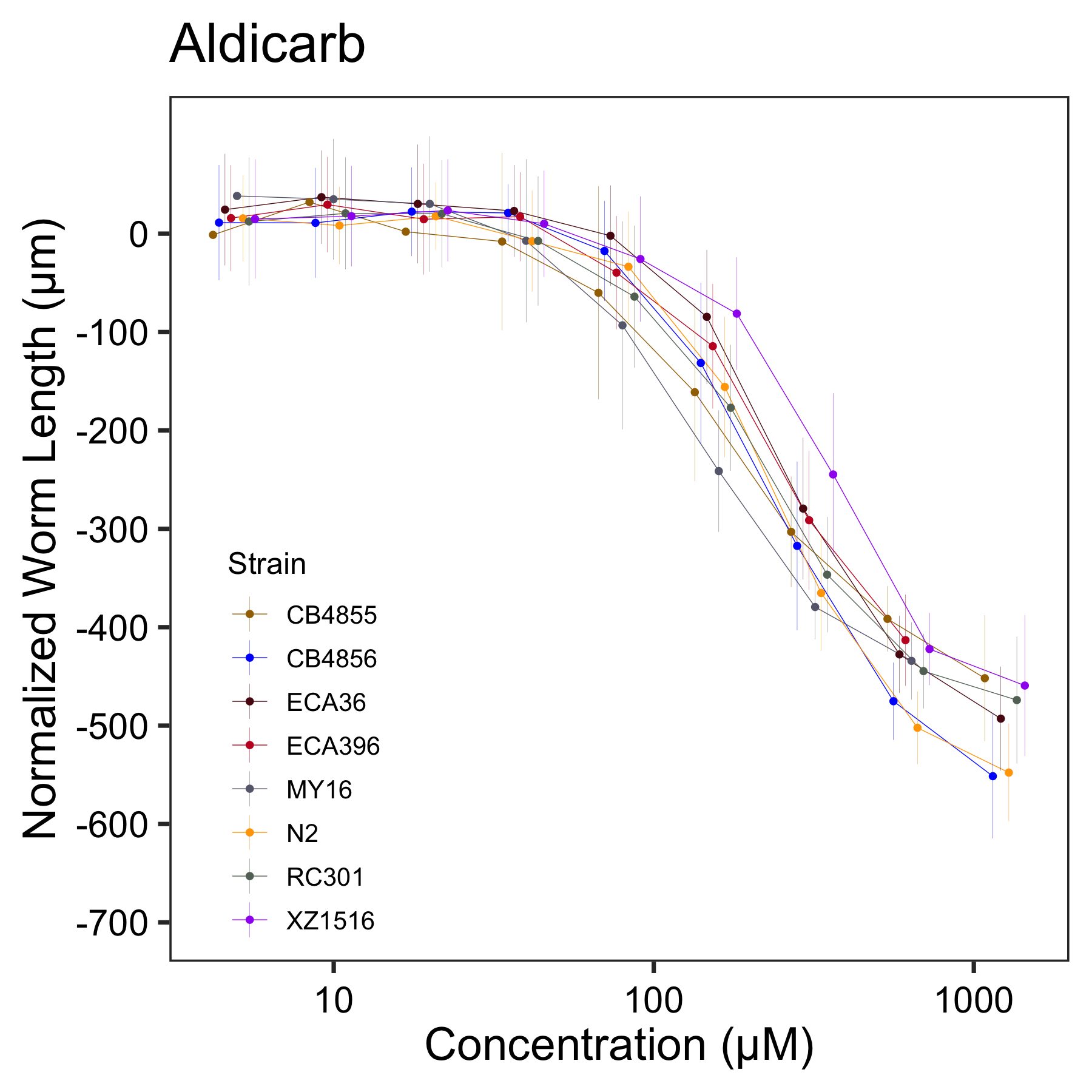

Supplement: SupFigs6-30 [file NIHMS1838727-supplement-SupFigs6-30.zip › mmc7/supp.fig.13.png]

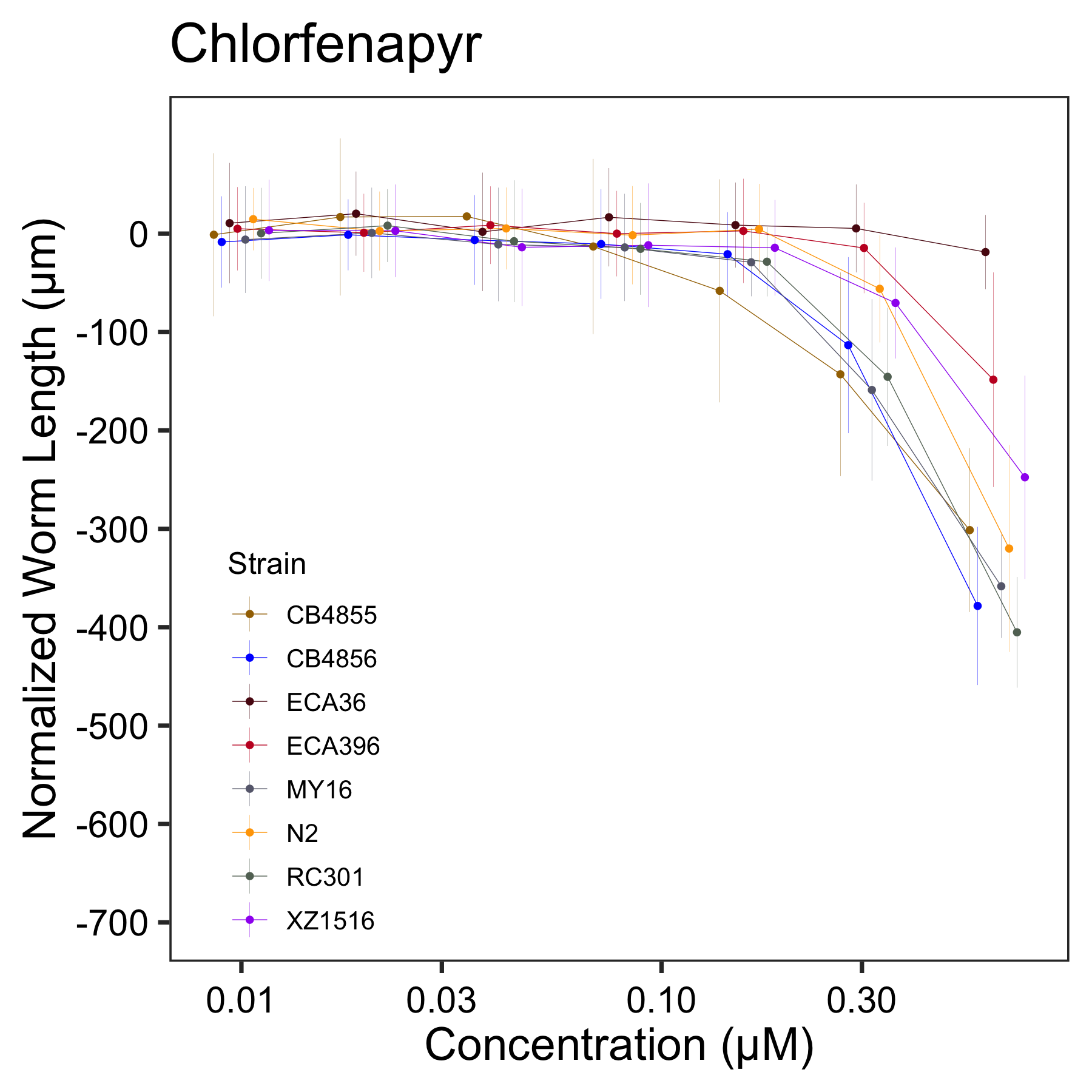

Supplement: SupFigs6-30 [file NIHMS1838727-supplement-SupFigs6-30.zip › mmc7/supp.fig.14.png]

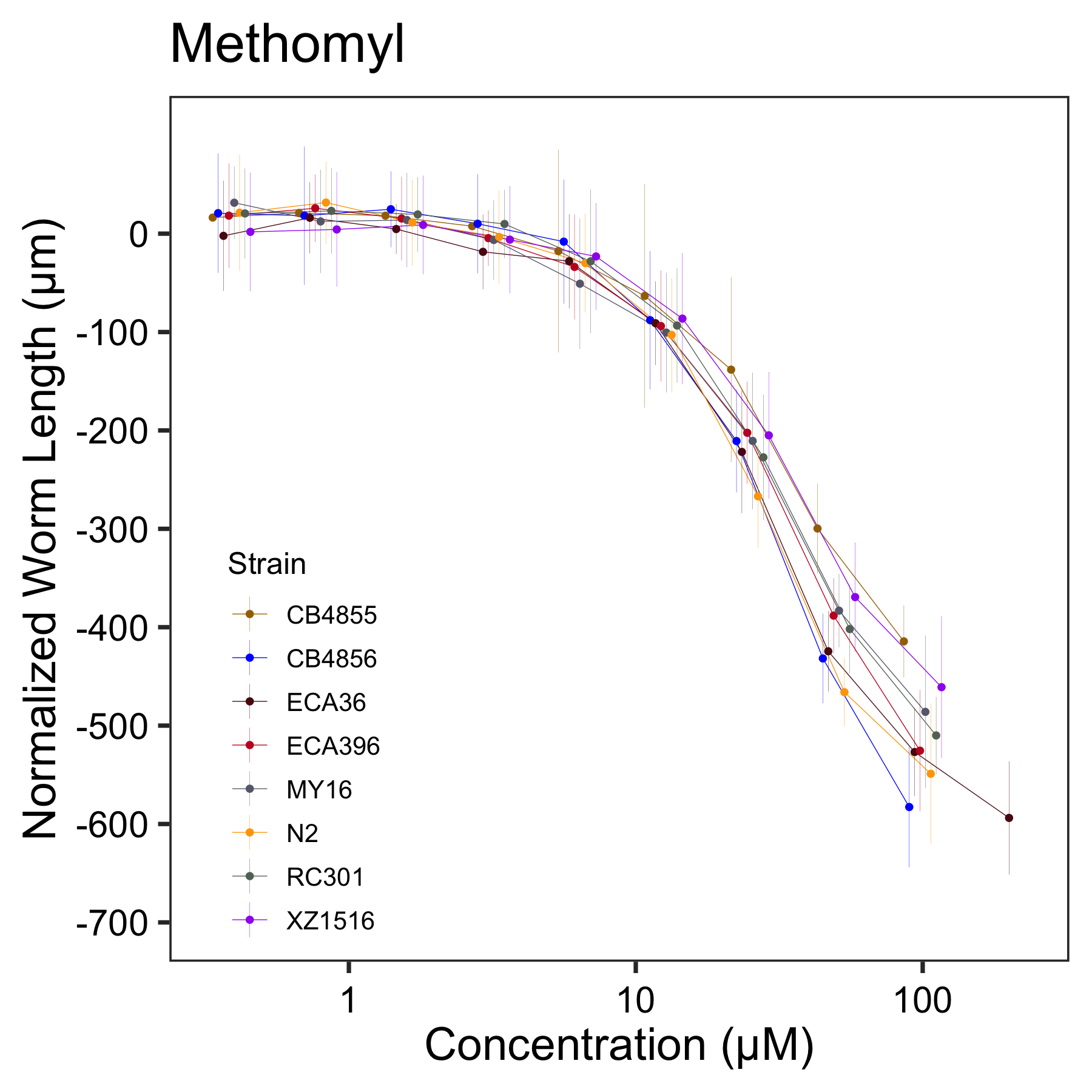

Supplement: SupFigs6-30 [file NIHMS1838727-supplement-SupFigs6-30.zip › mmc7/supp.fig.15.png]

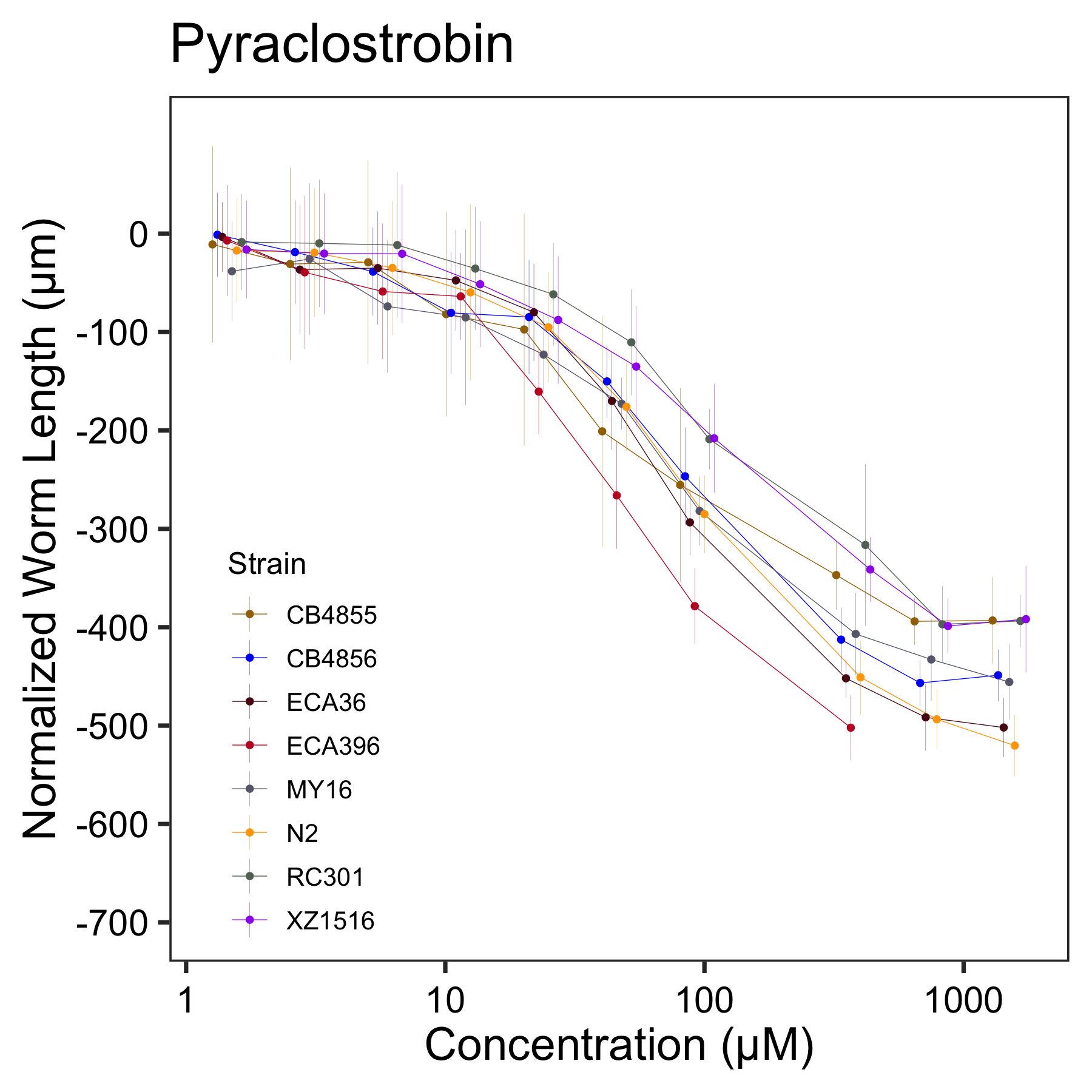

Supplement: SupFigs6-30 [file NIHMS1838727-supplement-SupFigs6-30.zip › mmc7/supp.fig.16.png]

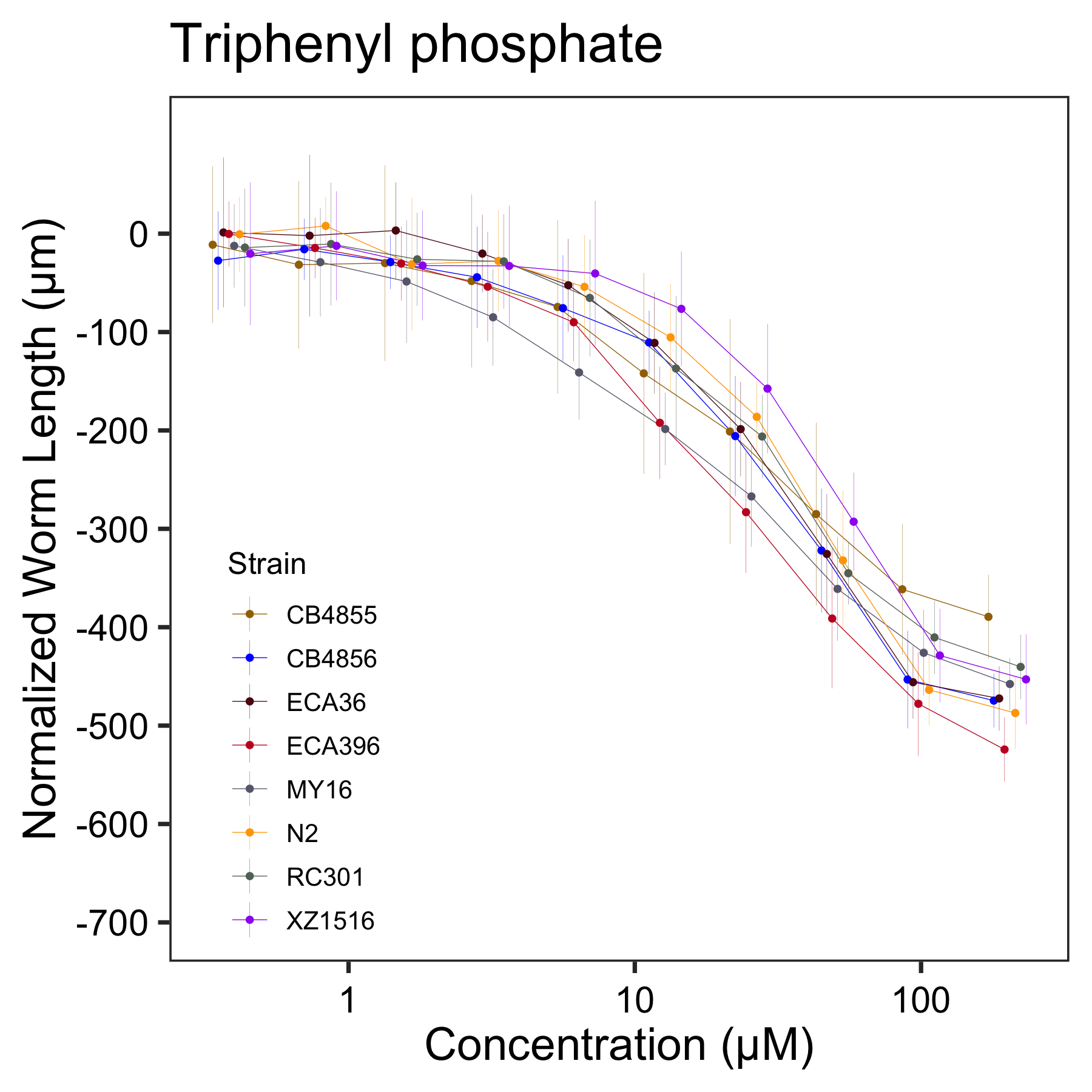

Supplement: SupFigs6-30 [file NIHMS1838727-supplement-SupFigs6-30.zip › mmc7/supp.fig.17.png]

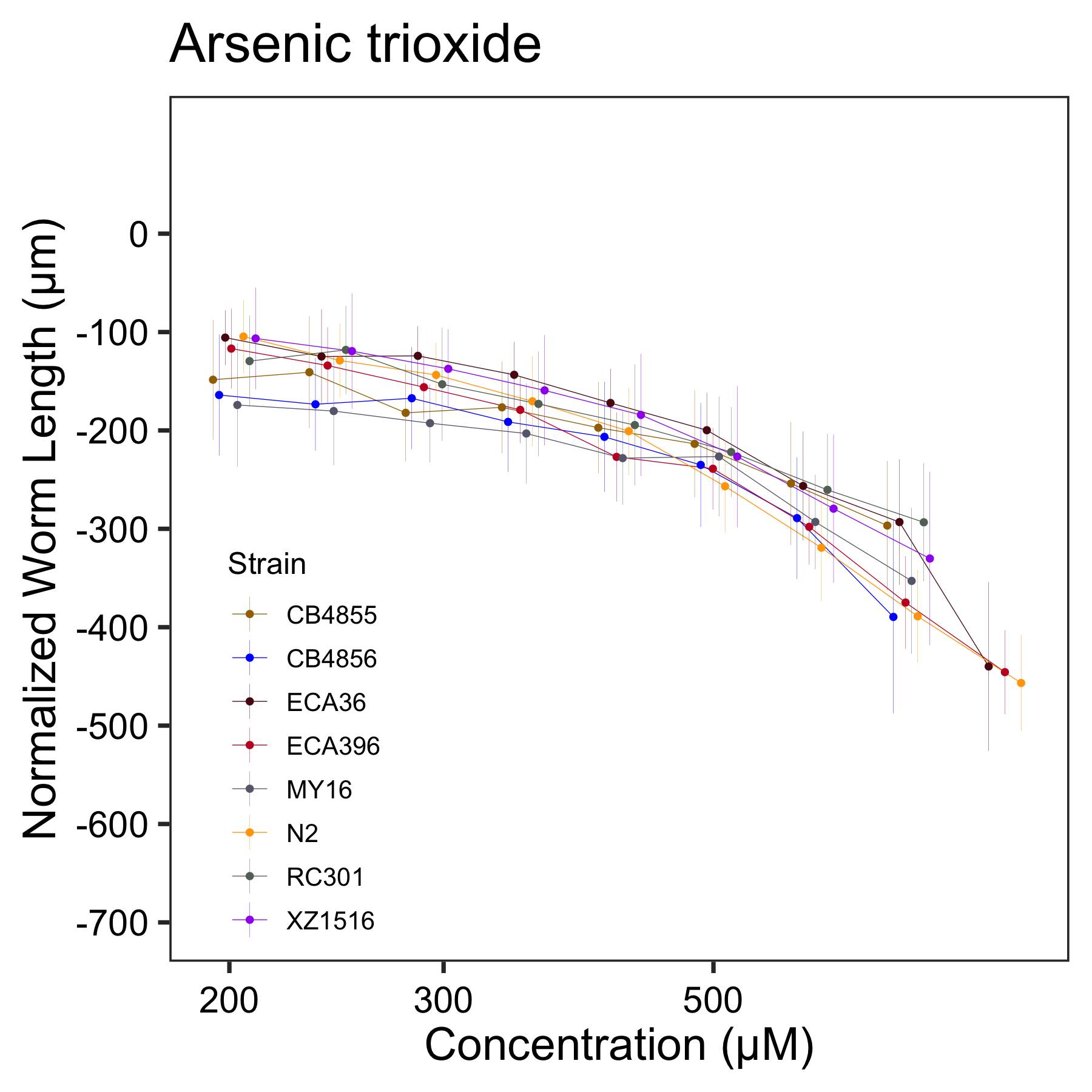

Supplement: SupFigs6-30 [file NIHMS1838727-supplement-SupFigs6-30.zip › mmc7/supp.fig.18.png]

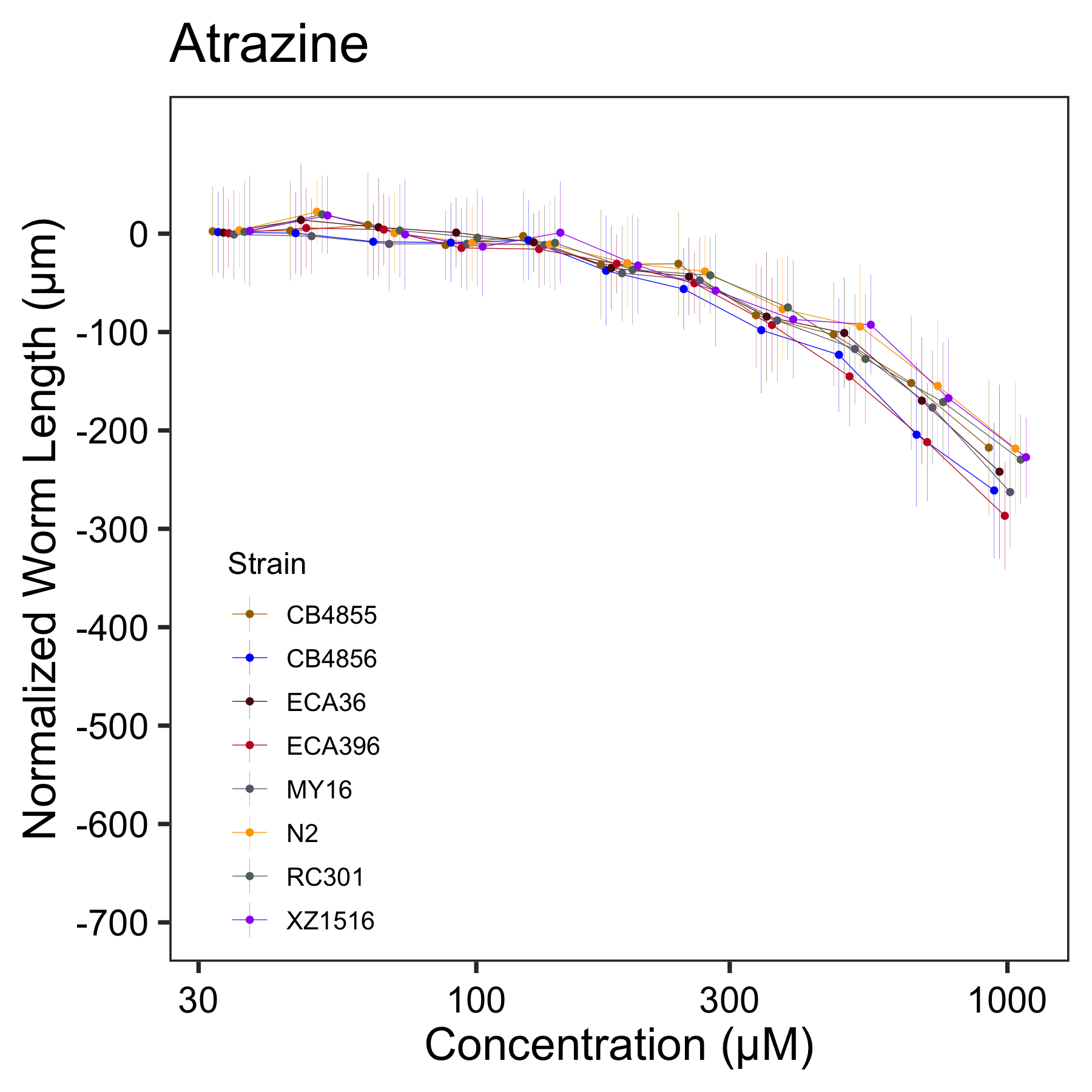

Supplement: SupFigs6-30 [file NIHMS1838727-supplement-SupFigs6-30.zip › mmc7/supp.fig.19.png]

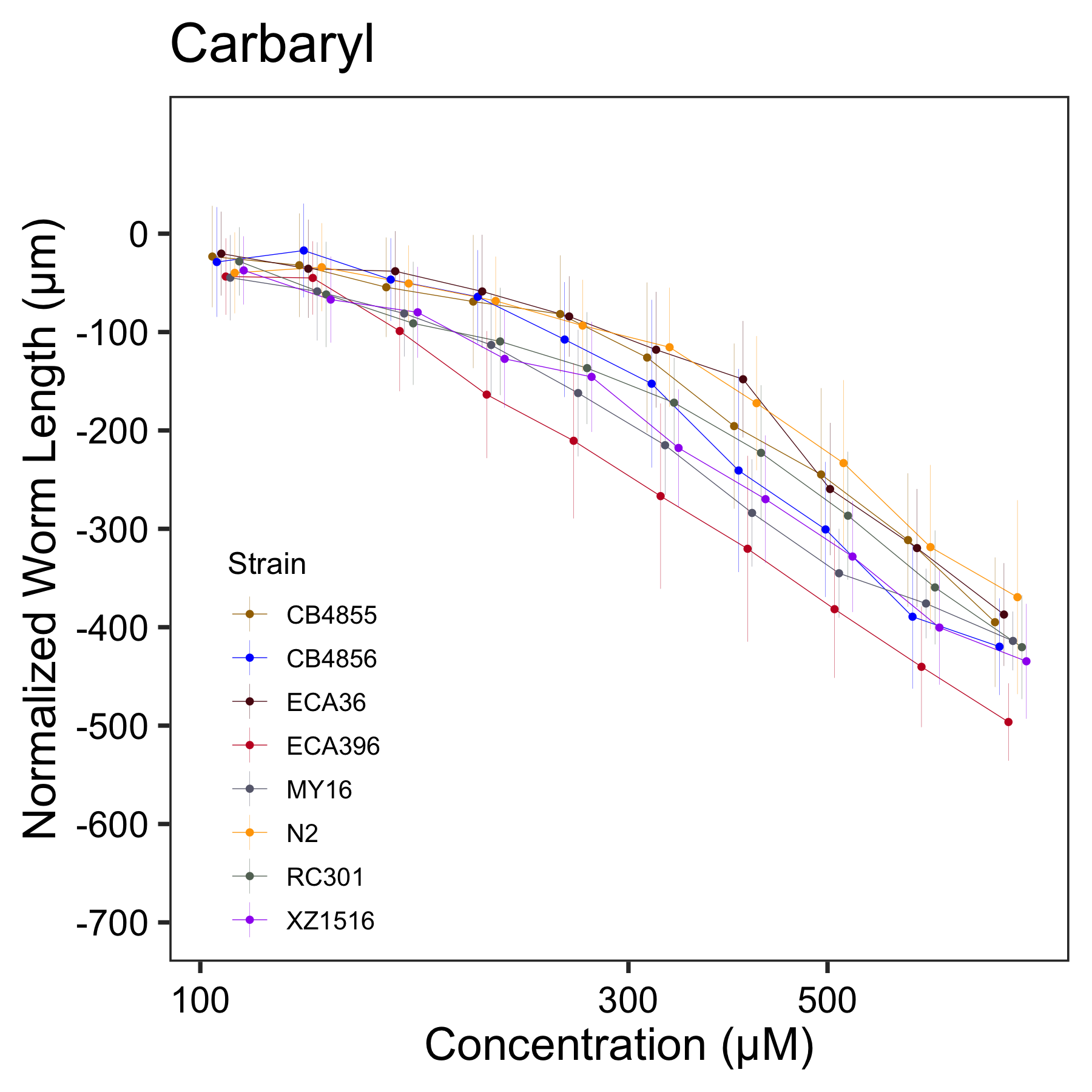

Supplement: SupFigs6-30 [file NIHMS1838727-supplement-SupFigs6-30.zip › mmc7/supp.fig.20.png]

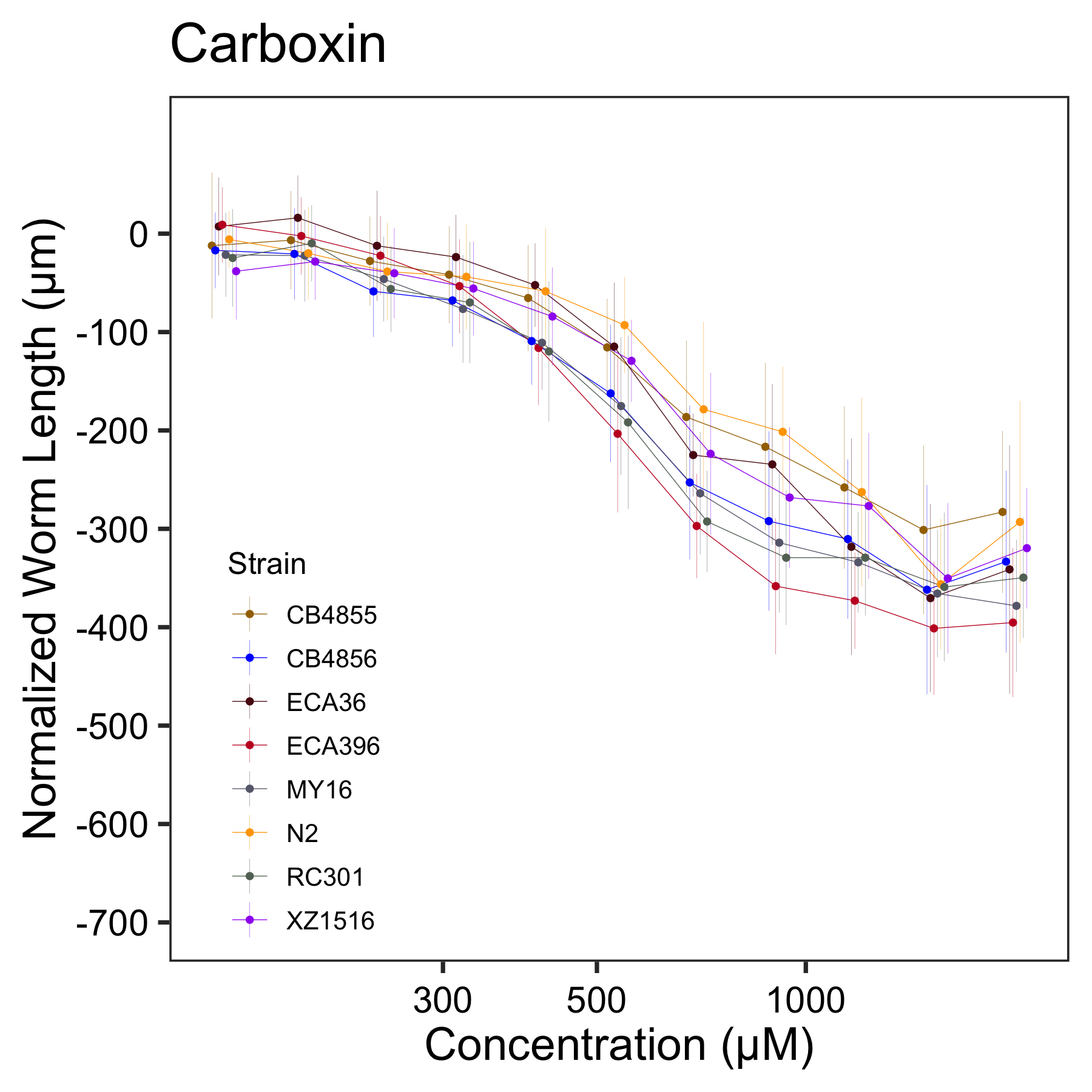

Supplement: SupFigs6-30 [file NIHMS1838727-supplement-SupFigs6-30.zip › mmc7/supp.fig.21.png]

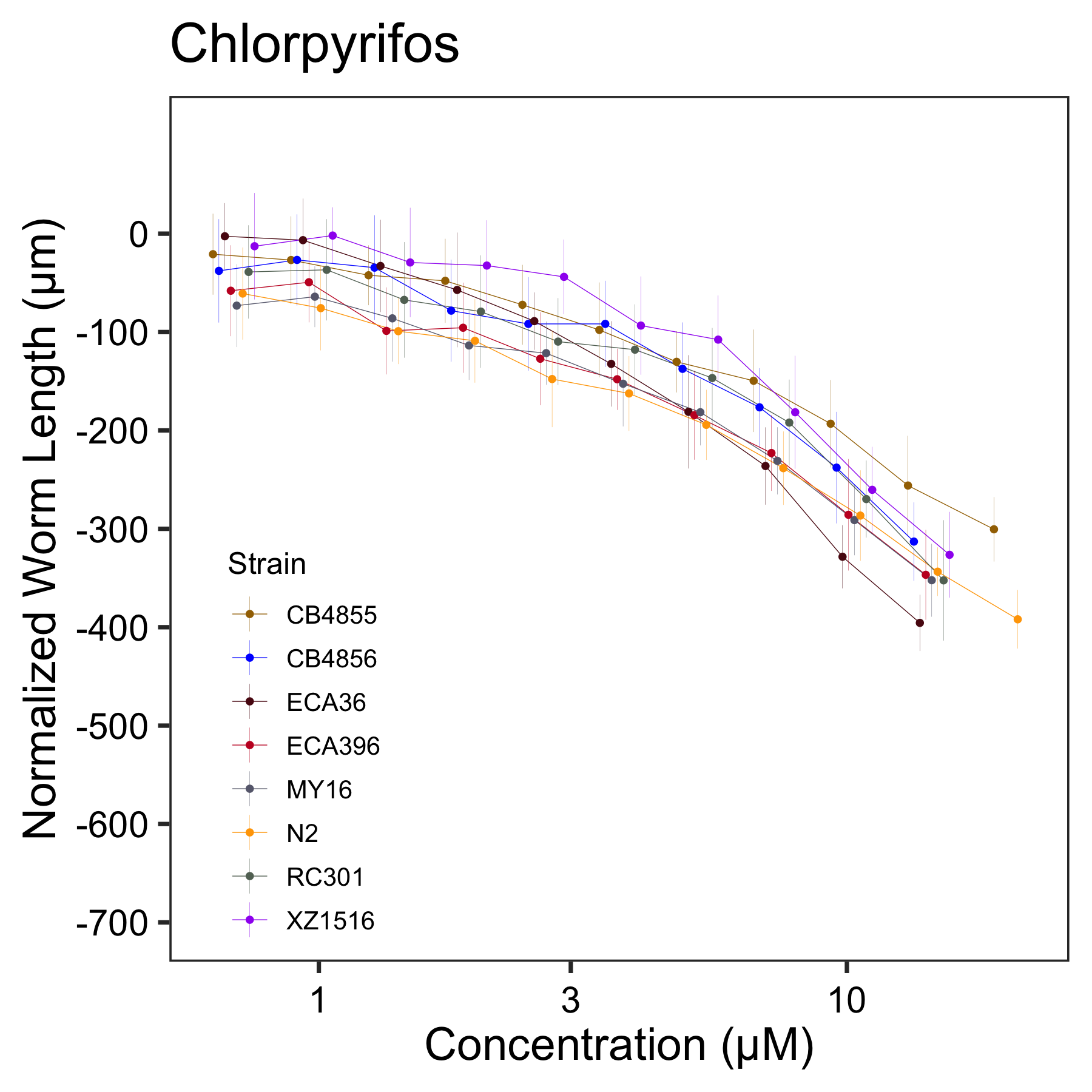

Supplement: SupFigs6-30 [file NIHMS1838727-supplement-SupFigs6-30.zip › mmc7/supp.fig.22.png]

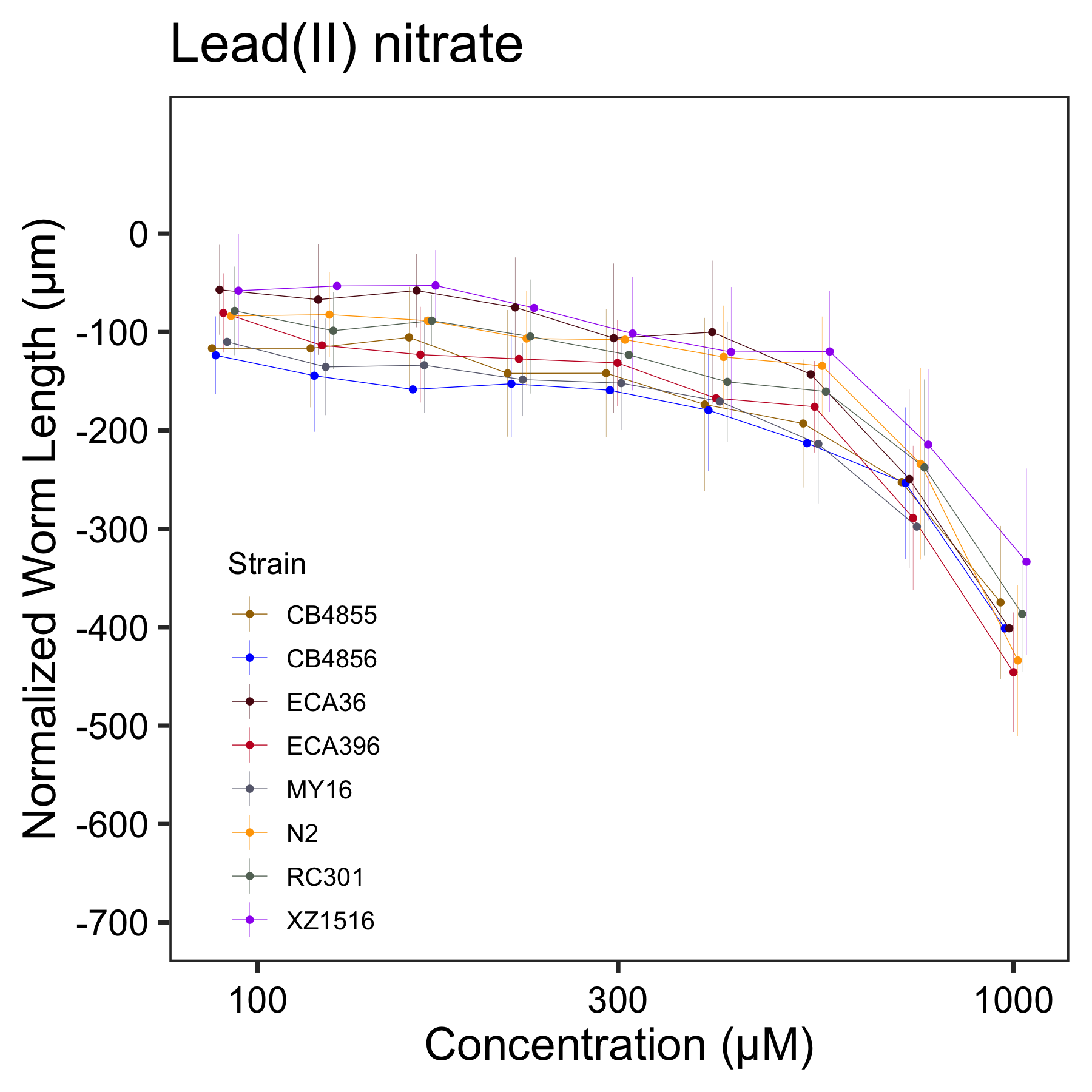

Supplement: SupFigs6-30 [file NIHMS1838727-supplement-SupFigs6-30.zip › mmc7/supp.fig.23.png]

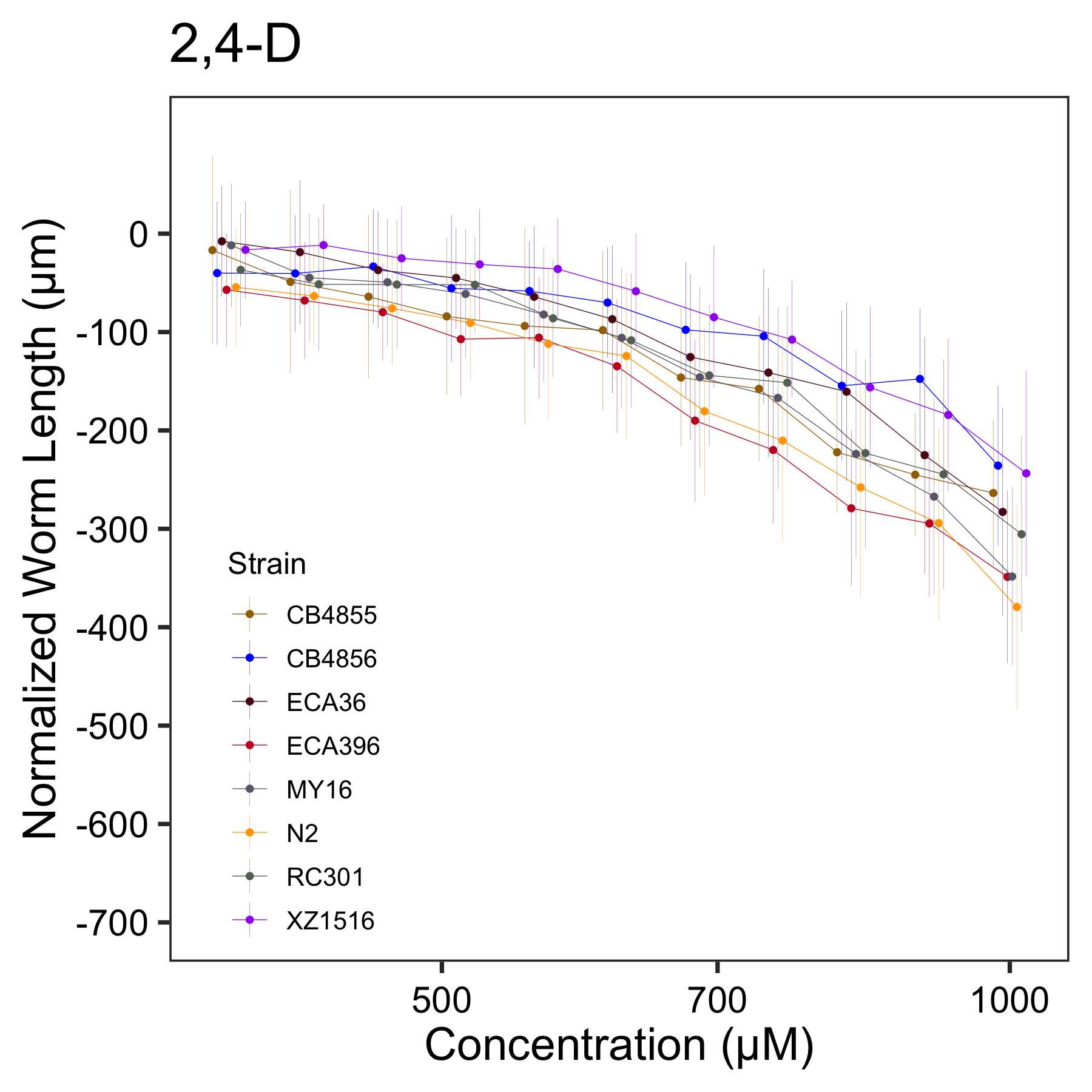

Supplement: SupFigs6-30 [file NIHMS1838727-supplement-SupFigs6-30.zip › mmc7/supp.fig.24.png]

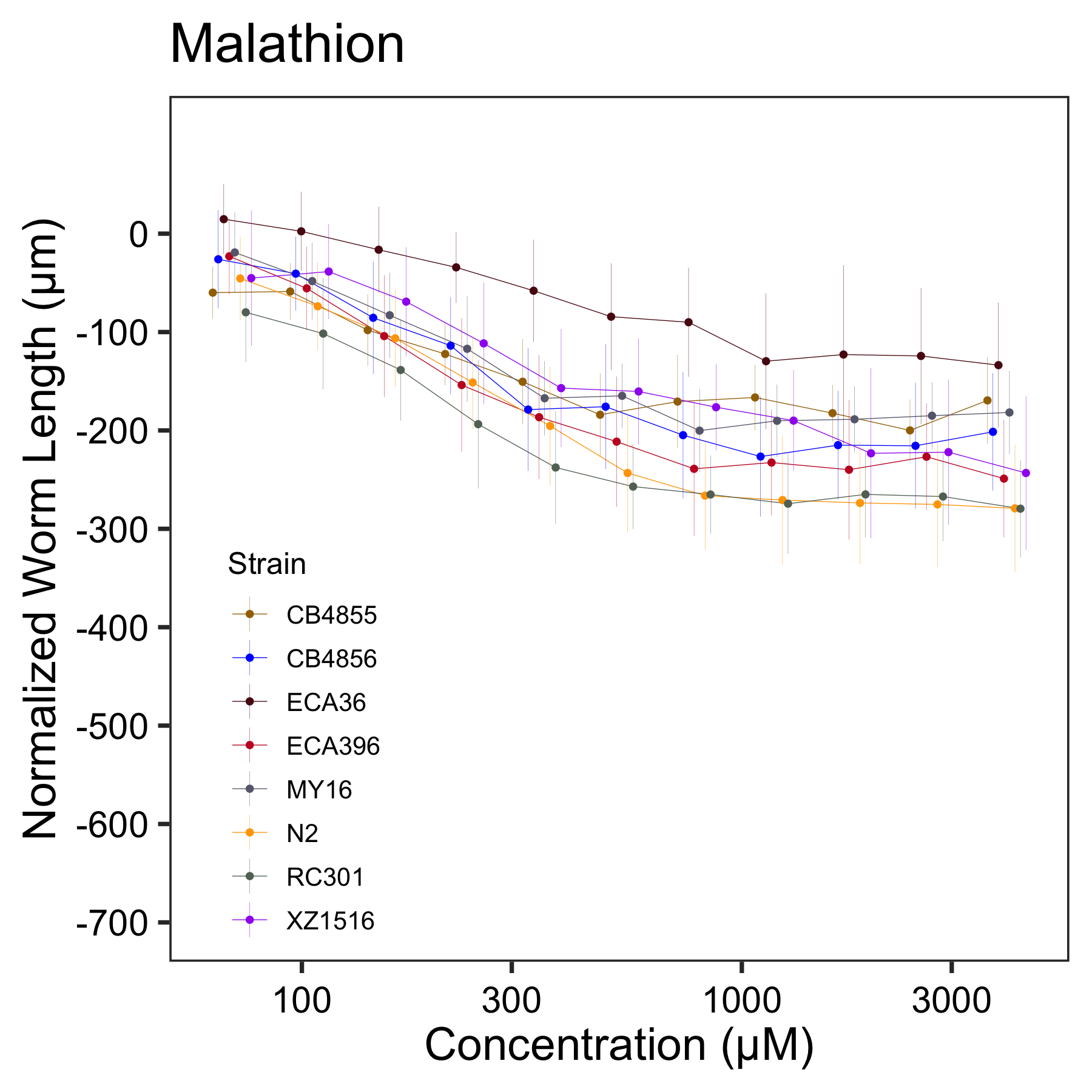

Supplement: SupFigs6-30 [file NIHMS1838727-supplement-SupFigs6-30.zip › mmc7/supp.fig.25.png]

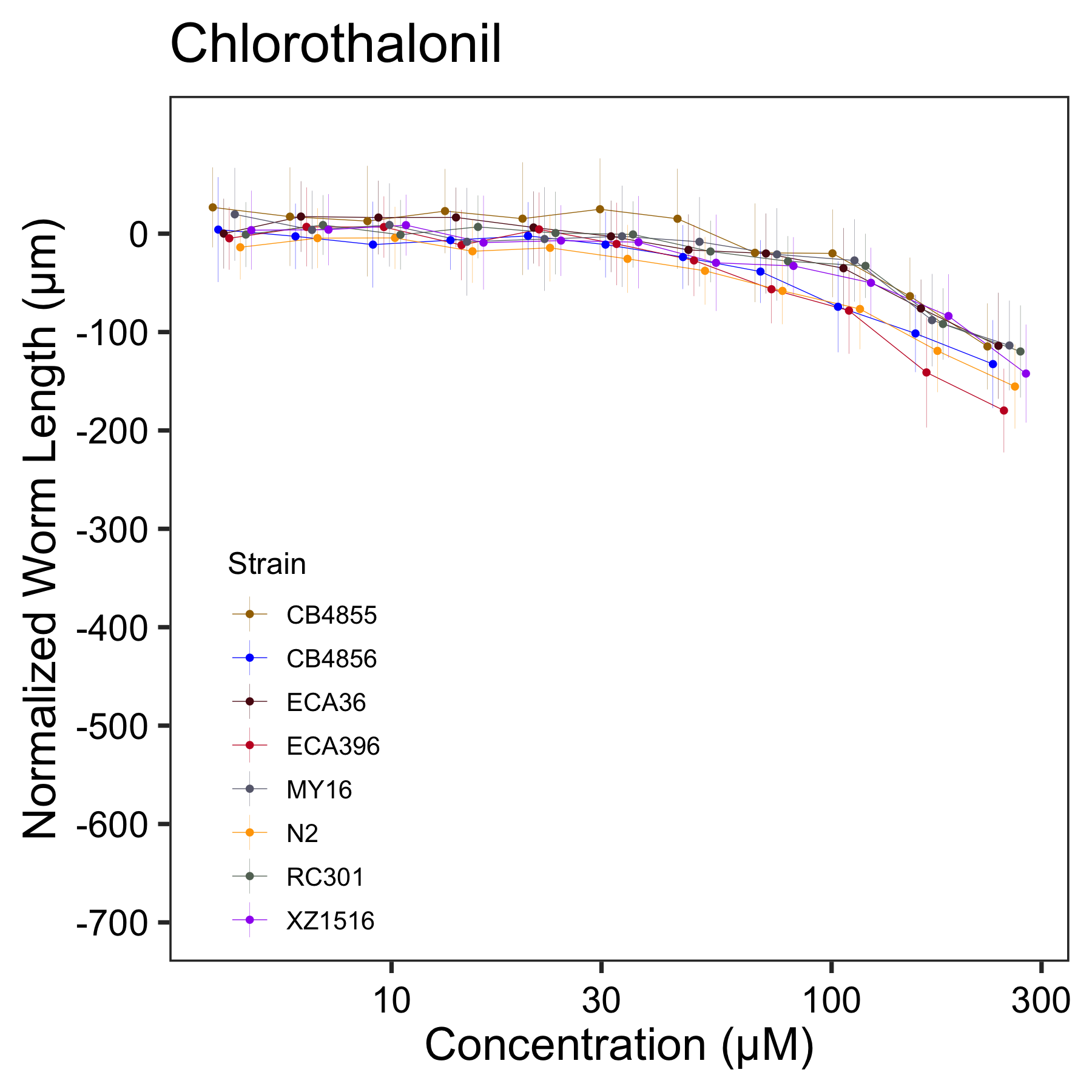

Supplement: SupFigs6-30 [file NIHMS1838727-supplement-SupFigs6-30.zip › mmc7/supp.fig.26.png]

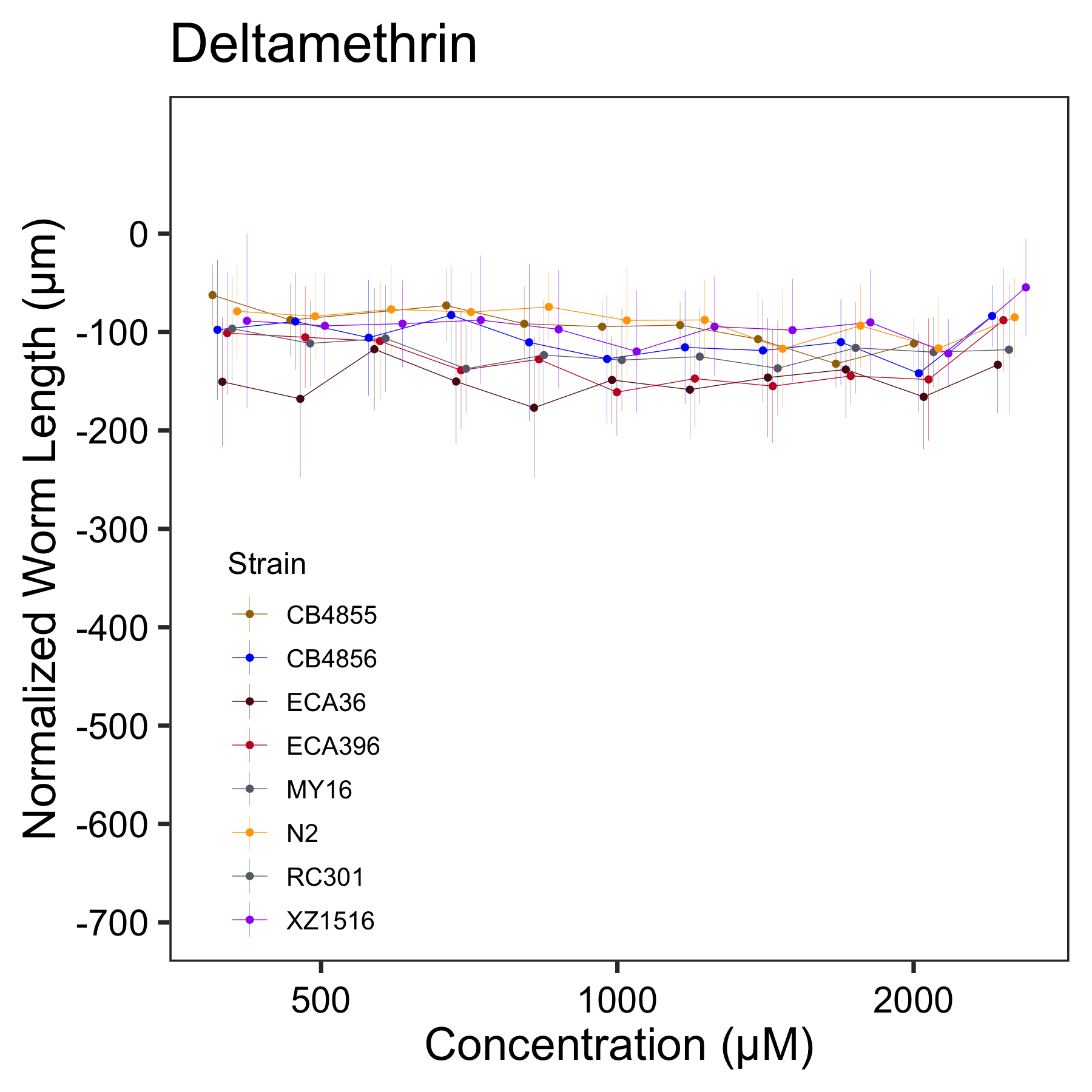

Supplement: SupFigs6-30 [file NIHMS1838727-supplement-SupFigs6-30.zip › mmc7/supp.fig.27.png]

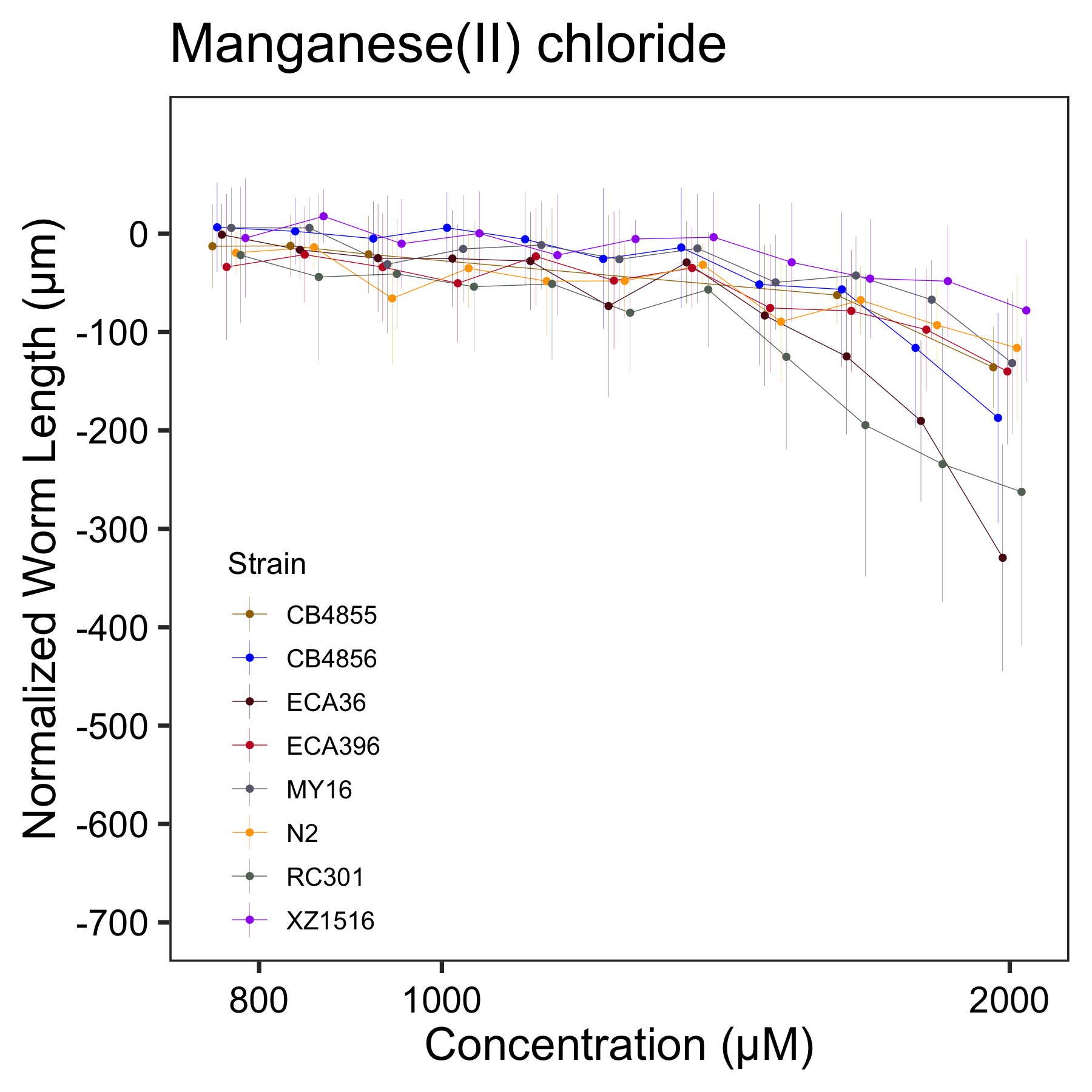

Supplement: SupFigs6-30 [file NIHMS1838727-supplement-SupFigs6-30.zip › mmc7/supp.fig.28.png]

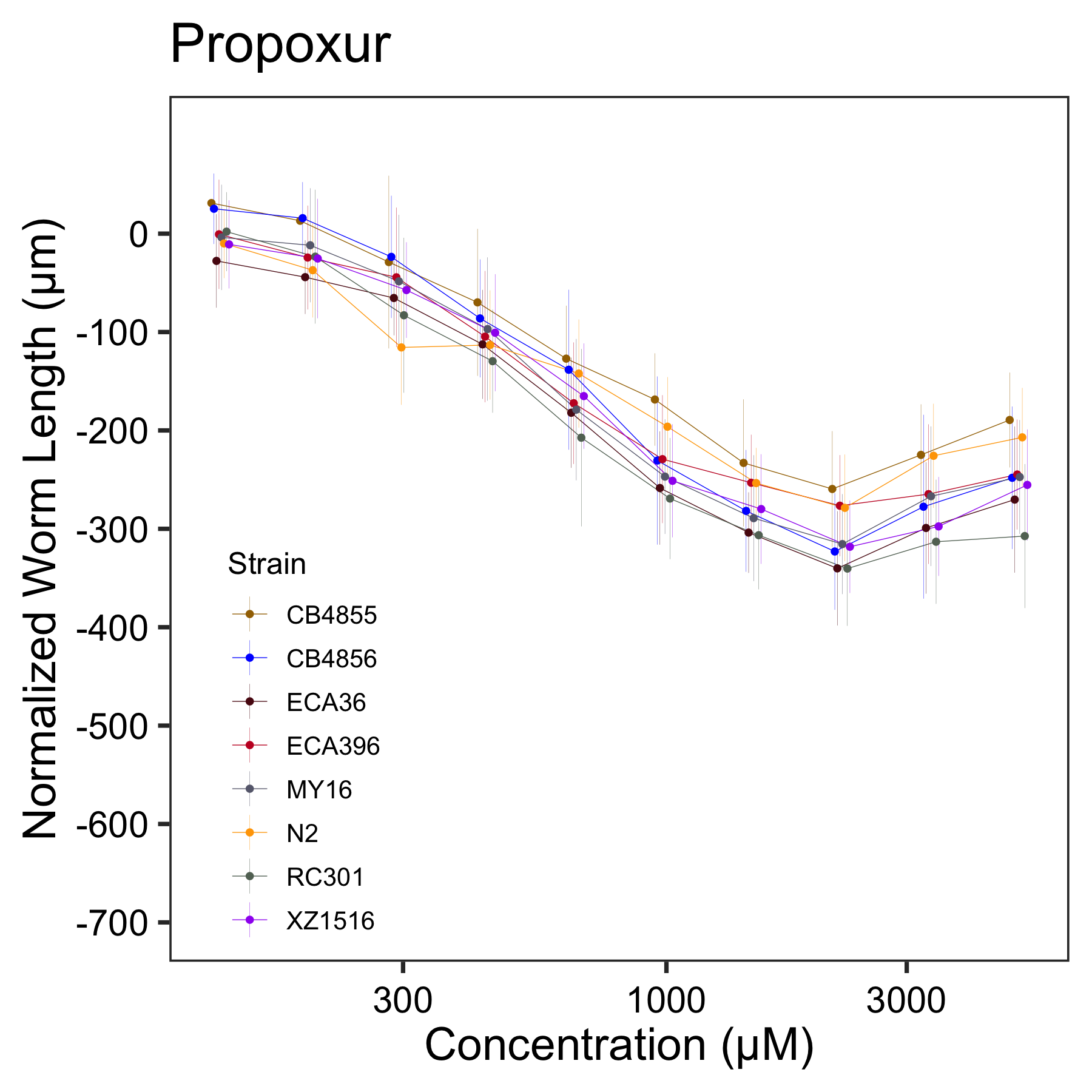

Supplement: SupFigs6-30 [file NIHMS1838727-supplement-SupFigs6-30.zip › mmc7/supp.fig.29.png]

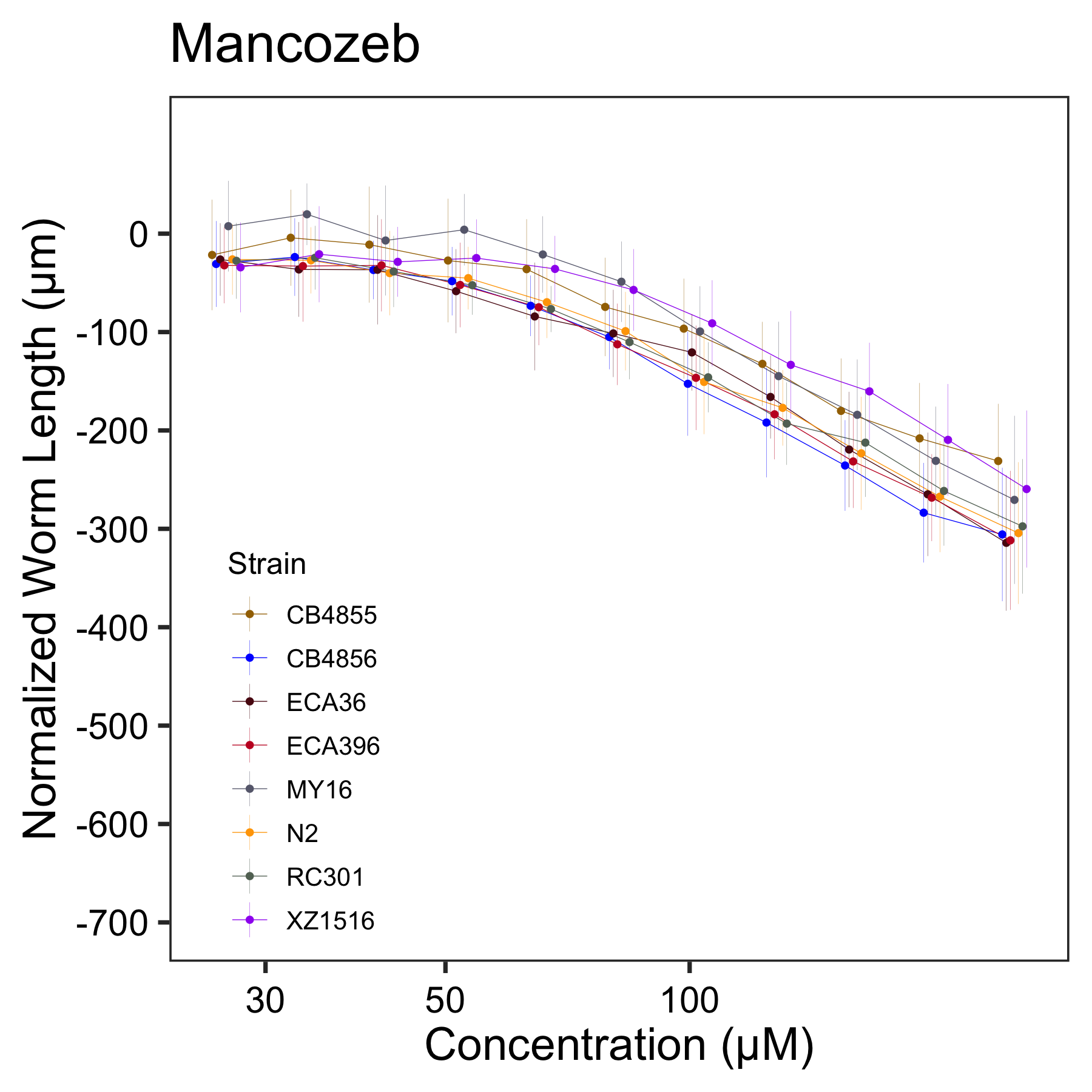

Supplement: SupFigs6-30 [file NIHMS1838727-supplement-SupFigs6-30.zip › mmc7/supp.fig.30.png]

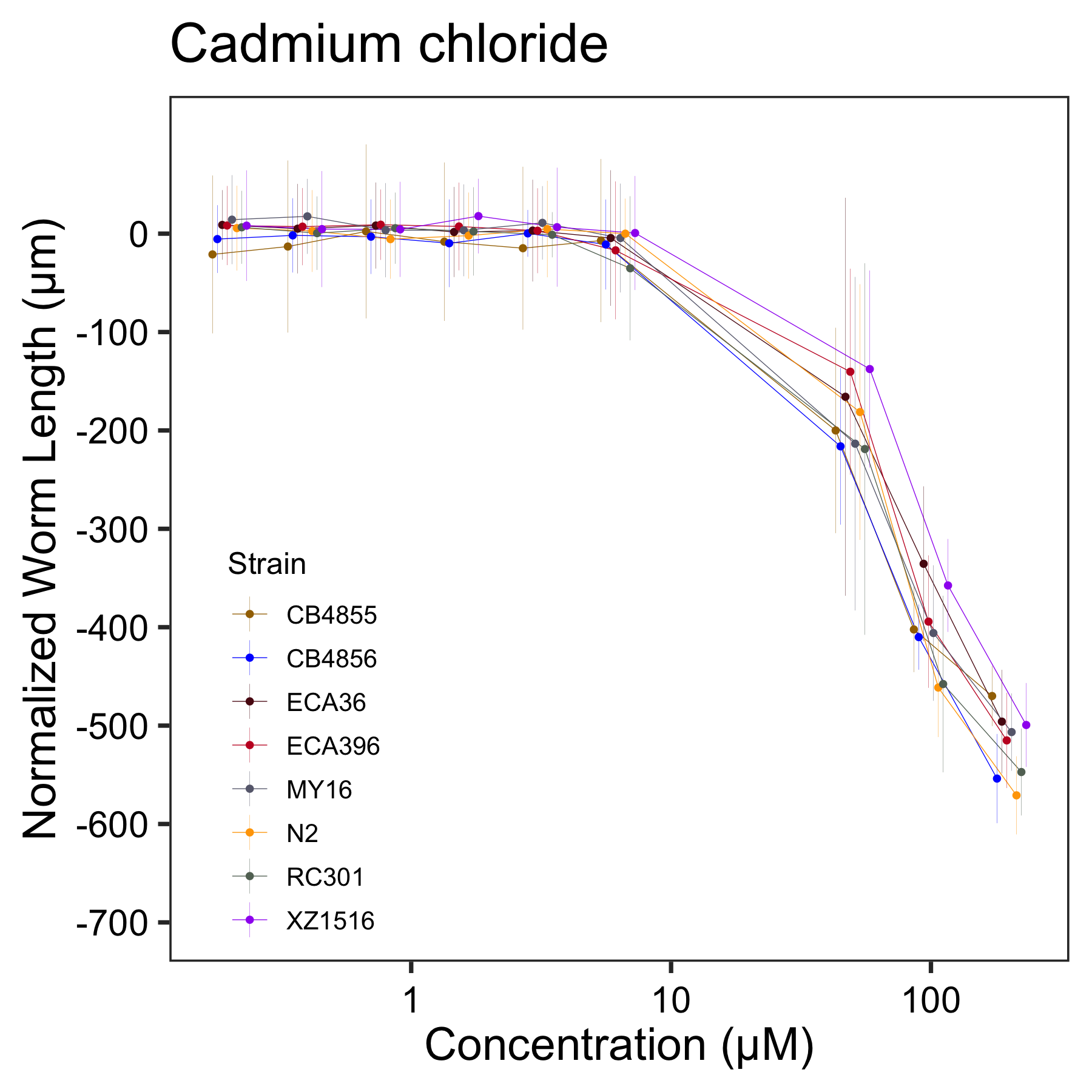

Supplement: SupFigs6-30 [file NIHMS1838727-supplement-SupFigs6-30.zip › mmc7/supp.fig.6.png]

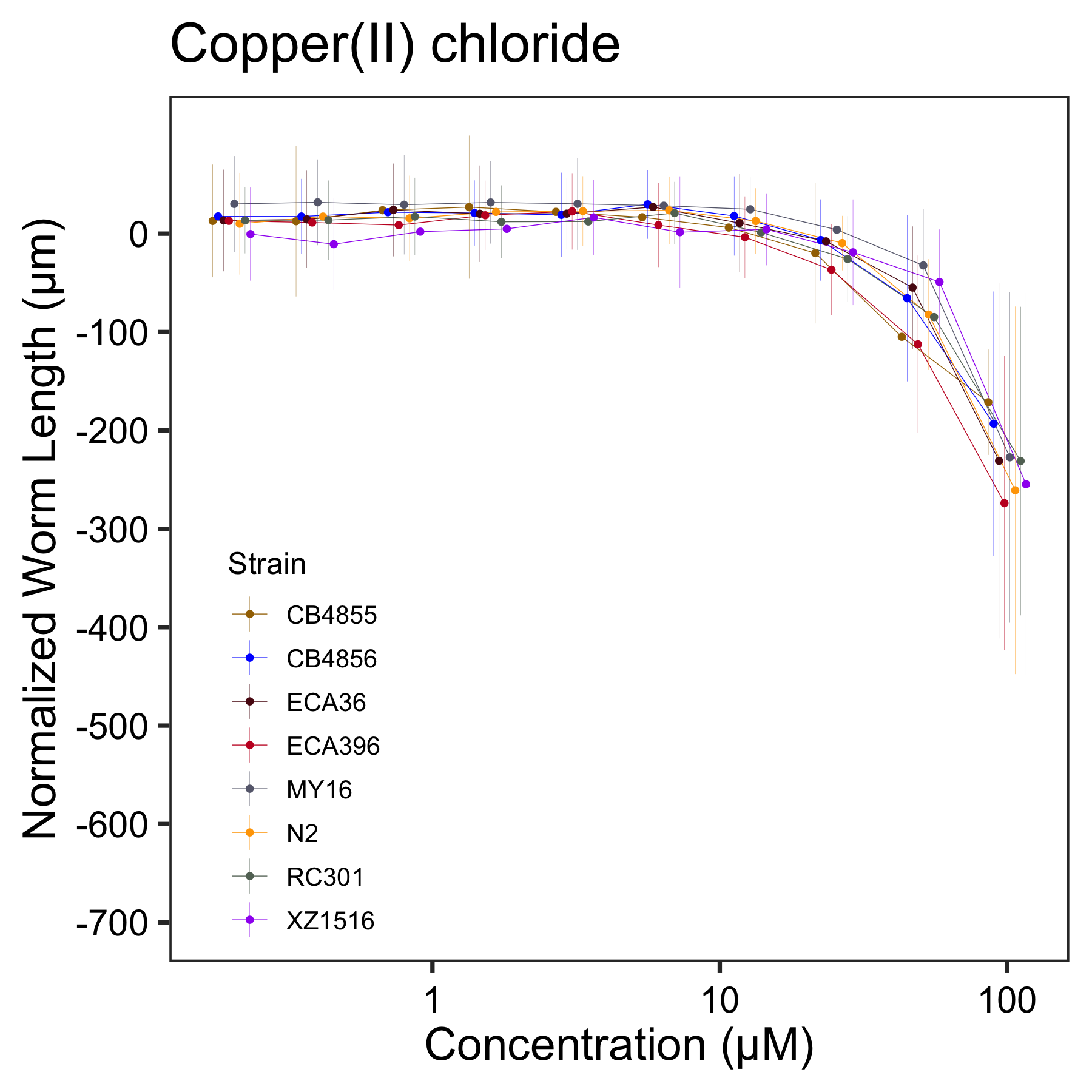

Supplement: SupFigs6-30 [file NIHMS1838727-supplement-SupFigs6-30.zip › mmc7/supp.fig.7.png]

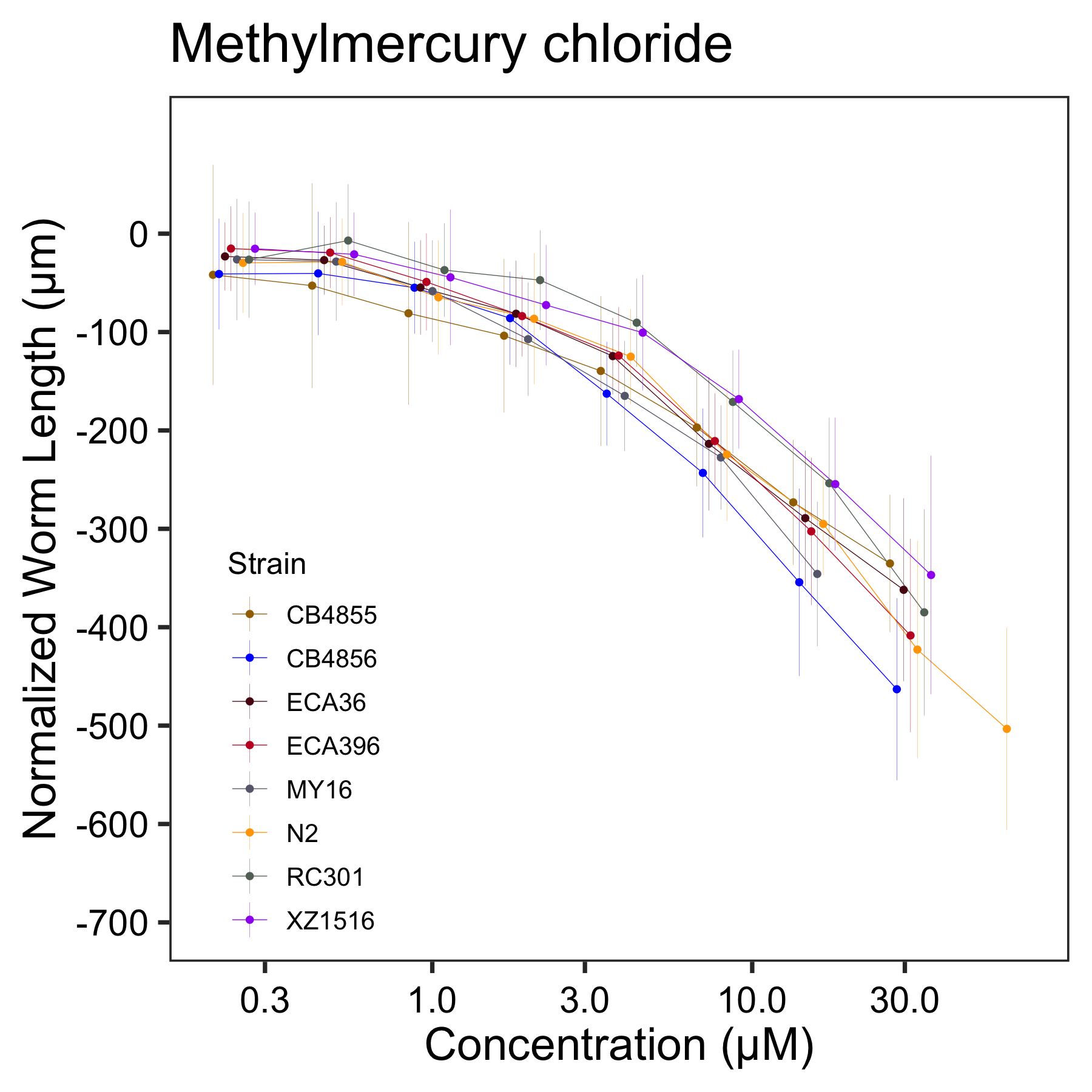

Supplement: SupFigs6-30 [file NIHMS1838727-supplement-SupFigs6-30.zip › mmc7/supp.fig.8.png]

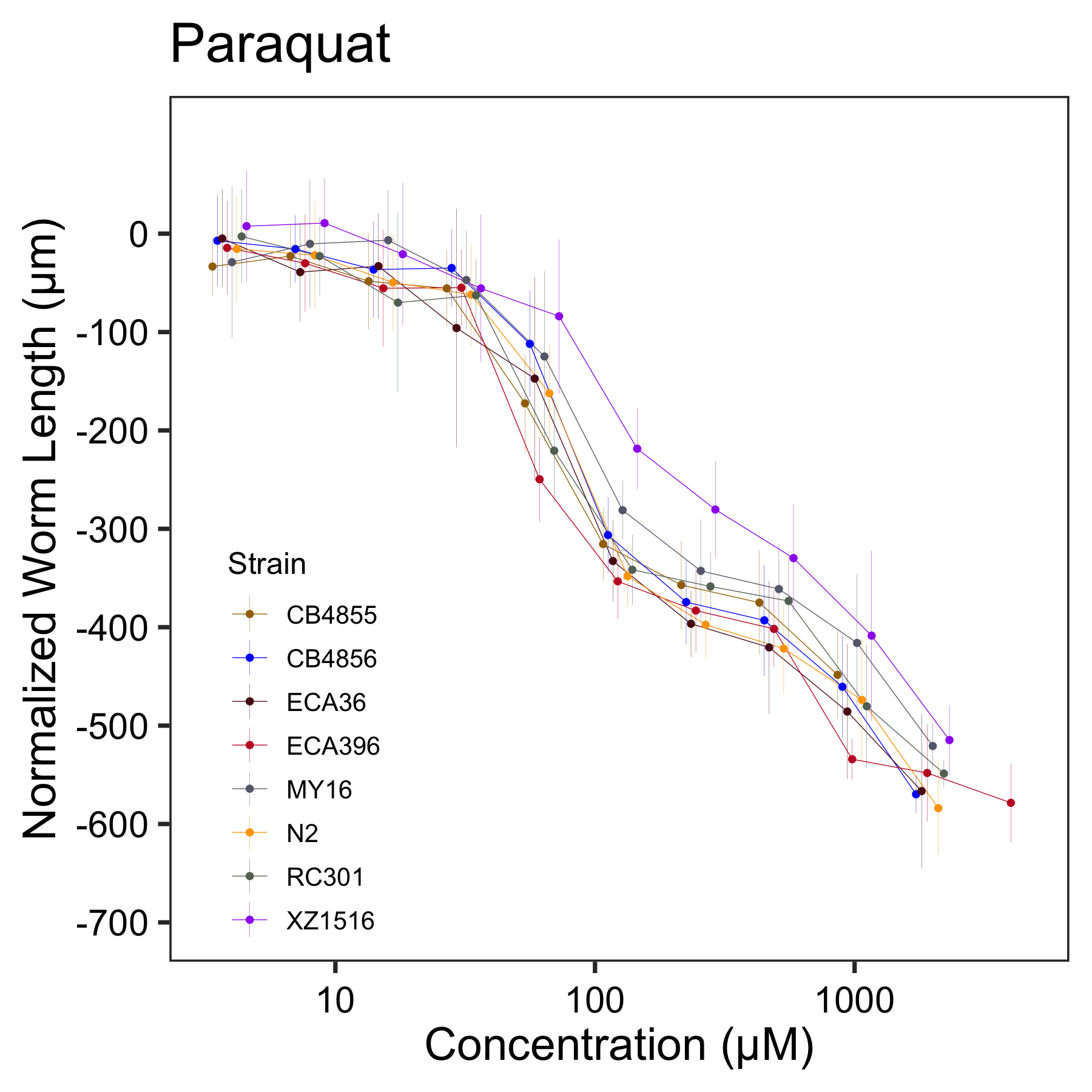

Supplement: SupFigs6-30 [file NIHMS1838727-supplement-SupFigs6-30.zip › mmc7/supp.fig.9.png]
